# *CFH* and *CFHR* Copy Number Variations in C3 Glomerulopathy and Immune Complex-Mediated Membranoproliferative Glomerulonephritis

**DOI:** 10.3389/fgene.2021.670727

**Published:** 2021-06-11

**Authors:** Rossella Piras, Matteo Breno, Elisabetta Valoti, Marta Alberti, Paraskevas Iatropoulos, Caterina Mele, Elena Bresin, Roberta Donadelli, Paola Cuccarolo, Richard J. H. Smith, Ariela Benigni, Giuseppe Remuzzi, Marina Noris

**Affiliations:** ^1^Istituto di Ricerche Farmacologiche Mario Negri IRCCS, Bergamo, Italy; ^2^Molecular Otolaryngology and Renal Research Laboratories, Carver College of Medicine, University of Iowa, Iowa City, IA, United States

**Keywords:** C3 glomerulopathy (C3G), immune complex-mediated membranoproliferative glomerulonephritis (IC-MPGN), factor H (FH), factor H-related proteins (FHRs), complement, copy number variations (CNVs), structural variants (SVs), single molecule real-time (SMRT)

## Abstract

C3 Glomerulopathy (C3G) and Immune Complex-Mediated Membranoproliferative glomerulonephritis (IC-MPGN) are rare diseases characterized by glomerular deposition of C3 caused by dysregulation of the alternative pathway (AP) of complement. In approximately 20% of affected patients, dysregulation is driven by pathogenic variants in the two components of the AP C3 convertase, complement C3 (*C3*) and Factor B (*CFB*), or in complement Factor H (*CFH*) and Factor I (*CFI*), two genes that encode complement regulators. Copy number variations (CNVs) involving the *CFH*-related genes (*CFHRs*) that give rise to hybrid FHR proteins also have been described in a few C3G patients but not in IC-MPGN patients. In this study, we used multiplex ligation-dependent probe amplification (MLPA) to study the genomic architecture of the *CFH-CFHR* region and characterize CNVs in a large cohort of patients with C3G (*n* = 103) and IC-MPGN (*n* = 96) compared to healthy controls (*n* = 100). We identified new/rare CNVs resulting in structural variants (SVs) in 5 C3G and 2 IC-MPGN patients. Using long-read single molecule real-time sequencing (SMRT), we detected the breakpoints of three SVs. The identified SVs included: 1) a deletion of the entire *CFH* in one patient with IC-MPGN; 2) an increased number of *CFHR4* copies in one IC-MPGN and three C3G patients; 3) a deletion from *CFHR3*-intron 3 to *CFHR3-3*′*UTR* (*CFHR3_4__–__6_*Δ) that results in a FHR3-FHR1 hybrid protein in a C3G patient; and 4) a *CFHR3_1__–__5_-CFHR4_10_* hybrid gene in a C3G patient. This work highlights the contribution of *CFH-CFHR* CNVs to the pathogenesis of both C3G and IC-MPGN.

## Introduction

Membranoproliferative glomerulonephritis (MPGN) is a heterogeneous group of rare glomerular diseases associated with complement dysregulation, which leads to the deposition of C3 and its cleavage products in glomeruli. Diagnosis requires a kidney biopsy, as the clinical presentation and course are variable, with patients manifesting asymptomatic haematuria and proteinuria, hypertension, nephritic or nephrotic syndrome, and/or acute kidney injury. Approximately 50% of patients develop chronic kidney disease (CKD) and progress to end-stage renal failure (ESRF) over a 10-year period (Sethi and Fervenza, 2011; Noris and Remuzzi, 2015). Current classification is based on glomerular deposits detected by immunofluorescence (IF) microscopy (Pickering et al., 2013). Cases with glomerular C3 staining in combination with significant immunoglobulin (IgGs) deposition are defined as immune-complex-mediated MPGN (IC-MPGN). C3 Glomerulopathy (C3G) is diagnosed in cases with dominant C3 staining at least two orders of magnitude greater than any other immunoreactant. Electron microscopy (EM) allows further differentiation of C3G into either dense deposit disease type (DDD), which is characterized by intramembranous highly electron-dense deposits, or C3 glomerulonephritis (C3GN), in which the deposits are less dense and have mesangial and/or subendothelial and subepithelial localization (Pickering et al., 2013; Hou et al., 2014).

**FIGURE 1:**
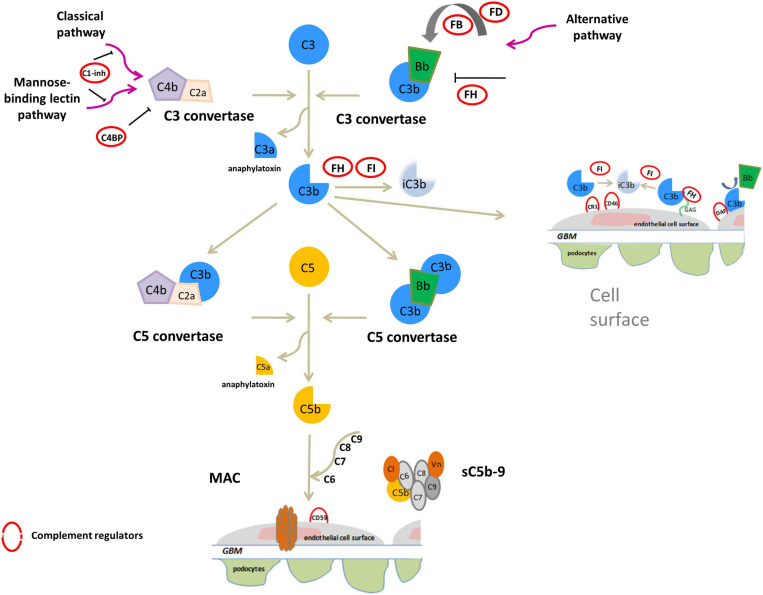
Overview of activation and regulation of complement system. The complement system is activated by three pathways: the classical (CP), the mannose-binding lectin (LP) and alternative (AP) pathways. All three activated cascades generate the C3 convertases (C4bC2a and C3bBb), proteolytic complexes that cleave C3 into C3a and C3b. C3a acts as an anaphylatoxin. C3b can covalently bind to surface membranes (e.g., intact host cells, microbial membranes, and modified host surfaces). Binding of an additional C3b molecule to C3 convertase generates C5 convertases (C4b2aC3b and C3bBbC3b) that cleave C5 into the potent anaphylatoxin C5a, and C5b. C5b recruits other complement components (C6, C7, C8, and C9) to assemble the soluble terminal C5b-9 complex (sC5b-9), which causes inflammation, or the membrane attack complex (MAC), leading to pore formation and target cell lysis. On healthy host cells the complement system is controlled at various steps by soluble or membrane regulators (indicated by red circles in the figure). FH binds to C3b and glycosaminoglycans (GAGs) on the cell surface and inactives C3b to iC3b in the presence of FI, and also accelerates the decay of the AP C3 convertase. C3b is inactivated to iC3b by FI also in the presence of membrane cofactor protein (CD46/MCP) or complement receptor 1 (CR1/CD35). In addition, among membrane complement regulators, DAF destabilizes and dissociates the C3/C5 convertases of the classical and alternative pathways while the CD59 (or protectin) binds C5b-8 complexes, inhibiting the recruitment of C9, thus preventing MAC generation. C1 inhibitor (C1-INH) and C4b-binding protein (C4BP) regulate the CP and LP. Vitronectin (Vn) and Clusterin (Cl) bind soluble C5b-7-8-9 complexes, blocking their incorporation into cell membranes.

Both C3G and IC-MPGN are complement-mediated diseases. The complement cascade is the cornerstone of innate immunity and can be initiated by three different pathways – the alternative (AP), classical (CP), or mannose-binding lectin (LP) pathways – that generate proteolytic complexes known as C3 convertases (Figure 1). The C3 convertase of the AP is C3bBb, while that of the CP and LP is C4bC2a. Both C3 convertases are so named because they cleave C3 into C3a, an anaphylatoxin, and C3b, which associates with factor B to generate additional C3bBb thereby amplifying the complement response. Binding of C3b to C3 convertases generates C5 convertases, which cleave C5 to produce C5a, another anaphylatoxin, and C5b, which initiates the terminal complement cascade by associating with other complement components (C6–C9) to form the terminal complement complex C5b-9 (Muller-Eberhard, 1986; Bhakdi and Tranum-Jensen, 1988; Morgan, 1999).

**FIGURE 2:**
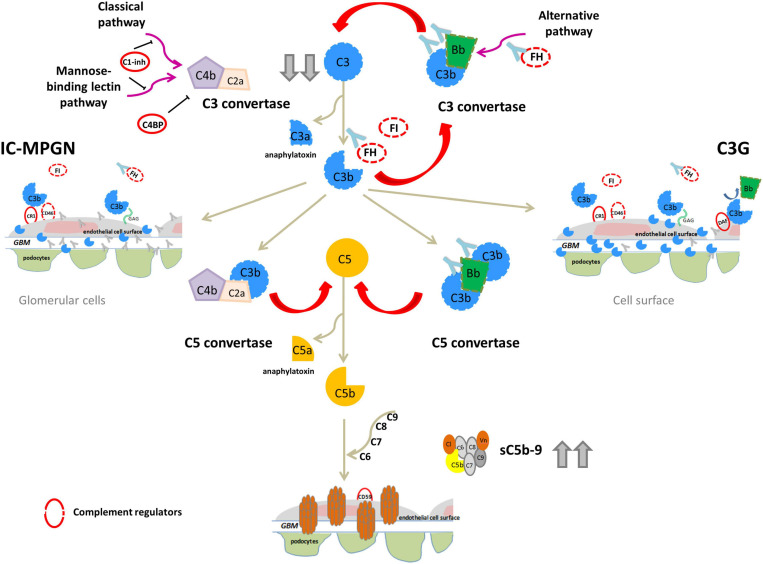
Complement dysregulation in C3G and IC-MPGN. Variants in complement alternative pathway (AP) genes (*CFH*, *C3*, *CFI*, and *CFB;* dashed lines) and/or autoantibodies (indicated by light blue Y-shaped forms) that bind FH, FB, C3b or that stabilize the C3/C5 convertase (nephritic factors, C3NeFs/C5NeFs) are the main drivers of complement AP dysregulation. This results in complement hyperactivation and glomerular deposition of C3 compounds (C3G). In some patients there is the concomitant activation of the classical pathway by infections or immune-complexes (IC) resulting in both C3 and IC deposits (IC-MPGN). In these patients AP dysregulation provides an activation loop exacerbating C3 glomerular deposition. In both cases, abnormal C3 convertase activation causes a consumption of circulating C3 that explains low serum levels in patients (indicated by gray arrows). Complement activation can proceed until the terminal pathway, causing high sC5b-9 plasma levels and glomerular C5b-9 deposits.

IC-MPGN has typically been linked to the activation of the complement CP following infections, autoimmune diseases or malignancies, while C3G has primarily been linked to activation of the complement AP (Sethi and Fervenza, 2011). In both C3G and IC-MPGN, genetic defects in complement AP genes like *CFH*, *C3*, *CFI*, and *CFB* (Servais et al., 2012; Iatropoulos et al., 2016, 2018), and acquired factors, such autoantibodies that stabilize the C3 convertase complex C3bBb (called C3-nephritic factors, C3NeFs) or against FH, FB and C3b have been identified (Zhang et al., 2012, 2020; Blanc et al., 2015; Marinozzi et al., 2017; Donadelli et al., 2018). These findings indicate that the dysregulation of the complement AP may underlie the pathogenesis of both diseases (Figure 2).

**FIGURE 3:**
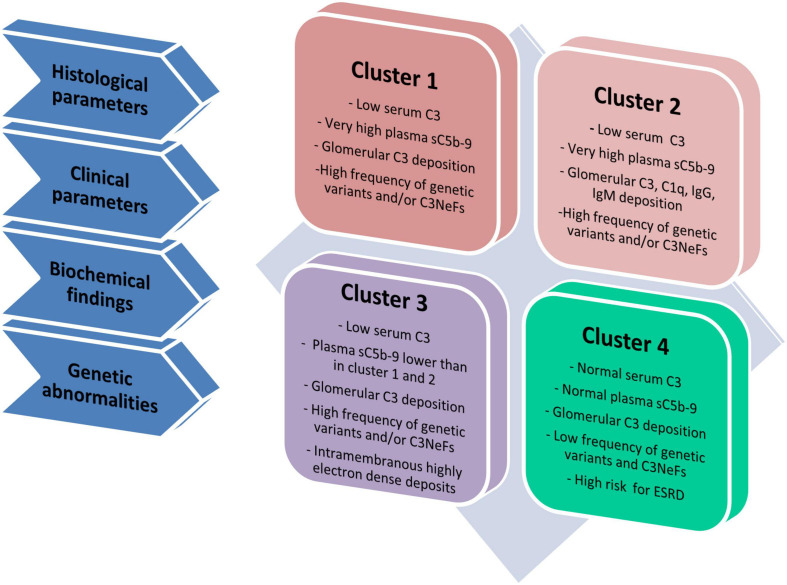
Schematic representation of four clusters. Cluster analysis was based on 34 variables, including histological, clinical, biochemical and genetic data and divided patients in four groups called clusters (Iatropoulos et al., 2018). Cluster 1, 2, and 3 have low C3 levels and high frequency of genetic variants and/or C3NeFs. Cluster 1 and 2 differentiate themselves from cluster 3 because of highly increased plasma levels of sC5b-9, indicative of high terminal pathway activity. Compared with cluster 1, cluster 2 includes patients with strong IgG, IgA and C1q glomerular deposition, indicating the concomitant activation of the classical pathway. At variance with cluster 1–3, cluster 4 is separated from the others, since it is characterized by normal C3 and sC5b-9 levels in face of intense glomerular C3 deposits, low frequency of genetic variants and/or C3NeFs and a high risk of developing end-stage renal disease (ESRD).

To gain further insights into the pathophysiology of these diseases, we have used unsupervised hierarchical cluster analysis based on histological, biochemical, genetic and clinical data at disease onset to divide our patient population into four clusters, each of which is defined by specific underlying pathophysiologic mechanisms (Figure 3) (Iatropoulos et al., 2018). In clusters 1, 2, and 3, serum C3 is low and the frequency of complement genetic variants and C3NeFs is high. Clusters 1 and 2 differentiated themselves from cluster 3 by very high sC5b-9 levels, which are indicative of dysregulated terminal pathway activity. Cluster 2 uniquely exhibits strong C1q, IgG and IgM glomerular deposition, suggesting that CP activity plays an important role in initiating disease in this cluster. Cluster 4 is characterized by normal C3 and sC5b-9 levels, and rare C3NeFs and complement genetic variants, despite intense C3 glomerular staining, indicating local glomerular complement activity (Iatropoulos et al., 2018).

Interestingly, genetic variants in *CFH*, which encodes factor H, the main regulatory protein of the AP complement pathway, are found in all 4 clusters indicating a complex pattern of functional consequences resulting in variable phenotypes (Iatropoulos et al., 2018).

The *CFH* gene family includes six genes - *CFH*, *CFHR3*, *CFHR1*, *CFHR4*, *CFHR2*, and *CFHR5*- on chromosome 1q31.3 that arose from *CFH* as a consequence of tandem genomic duplication events (Diaz-Guillen et al., 1999). The translated proteins, FH and FHR1-5s, are circulating proteins, organized in short consensus repeats (SCRs). The C-terminal region of the five *CFHRs* exhibits a high degree of sequence identity with the C-terminal domains of *CFH*, suggesting that FHR proteins can bind similar surface ligands as FH. However, FHRs do not contain the regulatory domains of FH (N-terminal region), suggesting they do not possess direct complement regulatory activity (Skerka et al., 2013).

The genomic region of the *CFH* gene family is characterized by large segmental duplications (SDs) and interspersed repetitive sequences that predispose to genomic rearrangements such as duplications, deletions and inversions (Lupski and Stankiewicz, 2005) that, when larger than 1kb, are called structural variants (SVs) (Feuk et al., 2006). The most common SV described in the *CFH* gene family is the ∼84 kb deletion of *CFHR3* and *CFHR1* (*CFHR3-CFHR1* del) with an allele frequency ranging from 2 to 51%, depending on ethnicity (Holmes et al., 2013). The absence of both copies of *CFHR3* and *CFHR1* is also associated with a lower risk of age-related macular degeneration (AMD) (Hughes et al., 2006) and IgA nephropathy (Gharavi et al., 2011), and a higher risk of atypical haemolytic uremic syndrome (aHUS) (Moore et al., 2010) and systemic lupus erythematosus (SLE) (Zhao et al., 2011).

Rare SVs involving *CFHRs* have been described in DDD and C3GN, most of which generate abnormal fusion proteins (Gale et al., 2010; Malik et al., 2012; Tortajada et al., 2013; Chen et al., 2014; Medjeral-Thomas et al., 2014; Togarsimalemath et al., 2017; Xiao et al., 2016). The classic example was identified in Greek Cypriot patients with C3GN (often called *CFHR5* nephropathy) that results from a mutant FHR5 protein encoded by a *CFHR5* gene with an internal duplication of exons 2 and 3 (FHR5_1_,_2_-FHR5) (Gale et al., 2010). Other FHR fusion proteins linked to C3G include FHR2_1_,_2_-FHR5 (Chen et al., 2014), FHR5_1_,_2_-FHR2 (Xiao et al., 2016), FHR1-FHR5 (Togarsimalemath et al., 2017), FHR1_1__–__4_-FHR1 (Tortajada et al., 2013), and FHR3_1_,_2_-FHR1 (Malik et al., 2012). These reports highlight the importance of the *CFH-CFHR* region in C3G and yet, with the exception of the fusion protein endemic to Cyprus, all fusion proteins thus far described have been identified in small families.

Comprehensive studies of the *CFH-CFHR* region in large cohorts of patients with C3G and IC-MPGN have not been reported. We sought to address this knowledge gap by identifying common and rare SVs and their distribution among the 4 C3G/IC-MPGN clusters we have described (Iatropoulos et al., 2018). SVs were detected using Multiplex Ligation-dependent Probe Amplification (MLPA) followed by PacBio long-read sequencing (SMRT, Single- Molecule Real-Time, sequencing) to provide base-pair resolution of selected genomic rearrangements.

## Materials and Methods

### Patients

Patients (*n* = 199) were recruited by the Italian Registry of MPGN, coordinated by the *Aldo e Cele Daccò* Clinical Research Center for Rare Diseases at the Mario Negri Institute. Clinical, demographic and laboratory data from patients were collected in a case report form. Blood, plasma and serum were also collected for biochemical and genetic tests. Controls included biological samples from blood donors (*n* = 214), which were analyzed for copy number abnormalities identified in C3G/IC-MPGN patients. The samples used for the research were stored at the Centro Risorse Biologiche (CRB) “Mario Negri”, biobank Malattie Rare e Malattie Renali.

The study was approved by the Ethics Committee of Bergamo (Italy). All participants received detailed information on the purpose and design of the study, according to the guidelines of the Declaration of Helsinki.

### Diagnosis

**FIGURE 4:**
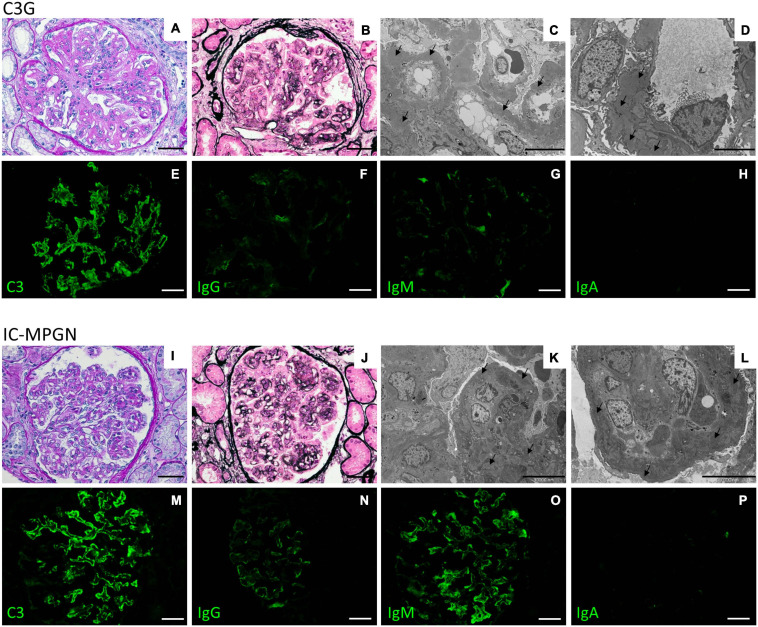
Representative biopsy findings from patients diagnosed with C3GN (upper panel) and IC-MPGN (lower panel). Upper panel: **(A,B)** C3GN patient. Light microscopy revealed endocapillary proliferation, leukocyte infiltration accompanied by lobulation of the glomerular tuft (**A**, periodic acid-Schiff staining; **B**, Jone’s silver staining). **(C,D)** Electron micrographs show intramembranous, subendothelial and mesangial deposits (arrows). **(E–H)** Representative immunofluorescence images reveal strong positivity for C3 staining along the capillary tuft **(E)**, while IgG **(F)**, IgM **(G)**, and IgA **(H)** staining is absent or present only in traces. (Scale bars: 50 μm in **A,B,E–H**; 10,000 nm in **C** and 5,000 in **D**). Lower panel: **(I–L)** IC-MPGN patient. Light microscopy and electron microscopy findings are similar to those observed in the C3GN patient (**I**, periodic acid-Schiff staining; **J**, Jone’s silver staining; **K,L**, transmission electron micrographs). **(M–P)** At variance with C3GN patients, immunofluorescence analysis shows the typical pattern of IC-MPGN, with abundant deposition of C3 in the glomerular tuft **(M)**, accompanied by moderate-to-strong positivity for IgG and IgM immunoglobulin staining (**N**, IgG; **O**, IgM); IgA staining is negative (**P**, IgA). (Scale bars: 50 μm in **I,J,M–P**; 10,000 nm in **K,L**).

All kidney biopsy reports were independently reviewed by two pathologists at the Mario Negri Institute and discordances were resolved through face-to-face discussion (Iatropoulos et al., 2018). The diagnosis of MPGN was based on light microscopy findings, according to the Cook HT and Pickering MC (Cook and Pickering, 2015). MPGN patients were further classified by immunofluorescence (IF) as (Sethi and Fervenza, 2011; Pickering et al., 2013; Iatropoulos et al., 2016; Marinozzi et al., 2017): (1) Immune-complex-mediated MPGN (IC-MPGN) – C3 and IgG IF similar or differing by less than two orders of magnitude; or, (2) C3 Glomerulopathy (C3G) – C3 IF at least two orders of magnitude greater than any other immune reactant (scale of 0 to 3) (Figure 4).

Based on electron microscopy (EM) findings, C3G was further classified as either DDD or C3GN. Patients with secondary MPGN, a previous diagnosis of aHUS, MPGN on allograft but without biopsy of native kidney, and without IF or EM studies, were excluded from this study.

All patients from the Registry who fulfilled the above inclusion criteria were included in this study.

### Cluster Analysis

We used a three-step algorithm to assign patients to different clusters, as reported in Iatropoulos et al. (2018). The algorithm is based on four features available at disease onset: genetic findings (presence of rare variants), C3NeF, serum C3 levels, biopsy findings (presence of intramembranous highly electron-dense deposits).

### DNA Samples

Genomic DNA (gDNA) was extracted from peripheral blood using either the Nucleon^TM^ BACC2 Genomic DNA extraction kit (GE Healthcare, Little Chalfont, United Kingdom) or NucleoSpin Blood columns (Macherey-Nagel). DNA integrity and quality were verified by 0.8% agarose gel electrophoresis and NanoDrop Spectometer (ND-1000; Thermo Fisher), respectively. Before genetic analyses, DNA was quantified using a Qubit fluorometer (dsDNA HS Assay kit; Invitrogen).

### Complement Component Assays

Serum C3 and C4 concentrations were measured by kinetic nephelometry (Noris et al., 2010). sC5b-9 levels were assessed using the MicroVue SC5b-9 Plus EIA commercial kit (SC5b-9 Plus, Quidel). IgGs purified from plasma were used to test C3NeF activity. The assay consisted in measuring IgG ability to stabilize the AP C3 convertase (C3bBb), as previously described (Fremeaux-Bacchi et al., 1994; Donadelli et al., 2018). The presence of anti-FH autoantibodies was evaluated by an Enzyme-Linked ImmunoSorbent Assay (ELISA), as reported (Valoti et al., 2019).

A in-house sandwich ELISA was developed to measure plasma or serum FH. In brief, Nunc MaxiSorp ELISA plates (Nunc, Roskilde, Denmark) were coated with 100 μL of diluted sheep polyclonal anti-factor H antibody (dilution 1:6333; Abcam) and were incubated overnight at 4°C. The next day, plates were washed with PBS and 0.05% Tween20, and blocked with PBS and 1% BSA for 1 h at RT. After washing, 100 μL of each diluted sample (1:10000 in PBS-BSA 1%) was added. After incubation for 2 h at RT, plates were washed with PBS and 0.05% Tween20. 100 μL mouse monoclonal anti-human Factor H (diluted 1:10000; OX-23, LS-C58560, LSBio), which specifically detects FH and FH-like (FHL1), was added to each well. After 2 h of incubation at RT, wells were washed and 100 μL of diluted goat anti-mouse IgG HRP conjugated (dilution 1:2000; Thermo Fischer Scientific) was added (1 h of incubation at RT). After washing, TMB was used as substrate to detect enzymatic activity. Enzymatic reactions were terminated using 100 μL of sulphuric acid and absorbance was read at 450 nm. All samples were tested in duplicate. Sample concentrations were extrapolated from sigmoidal curve. Serum/plasma samples of 102 healthy subjects were tested to establish normal FH levels (≥193 mg/L).

### Genetic Screening

Genetic analyses were performed by a next generation sequencing (NGS) diagnostic minipanel for simultaneous sequencing of 6 complement genes (complement factor H, *CFH*, NG_007259.1; complement factor I, *CFI*, NG_007569.1; membrane cofactor protein, *CD46/MCP*, NG_007569.1; complement factor B, *CFB*, NG_008191.1; complement C3, *C3*, NG_009557.1; and thrombomodulin, *THBD*, NG_012027.1). Amplicons were obtained by highly multiplex PCR using the Ion AmpliSeq^TM^ Library Kit 2.0 (Life Technologies, LT). Targets were then subjected to clonal amplification on Ion PGM^TM^ Template OT2 200 Kit and finally sequenced on Ion Torrent Personal Genome Machine Sequencer (PGM, LT), as previously described (Iatropoulos et al., 2016). In the patients with abnormal CNVs, we evaluated the presence of genetic variants in *CFHR1-5* by NGS studies, using either a panel called CasCADE, developed at the University of Iowa, or an updated version of the diagnostic minipanel (Bu et al., 2014).

Genetic variants in coding and splicing regions of complement genes with minor allele frequency (MAF) in the gnomAD database <0.001 and with a Combined Annotation Dependent Depletion (CADD) phred score ≥ 10 were considered rare variants (RVs). RVs were further classified into “pathogenic, (P)”, “likely pathogenic, (LPV),” and “variants of uncertain significance, (VUS)” using guidelines from the American College of Medical Genetics and Genomic (ACMG) and from the KDIGO conference on aHUS and C3G (Kircher et al., 2014; Richards et al., 2015; Goodship et al., 2017).

### Copy Number Variations (CNVs)

MLPA using the SALSA MLPA kit P236-A3 (MRC Holland) and in-house probes for *CFHR4* and *CFHR5* (Supplementary Table 1) were used to screen for rearrangements/deletions/duplications in the *CFH-CFHR5* genomic region in 199 patients (195 unrelated and 4 relatives) and in 100 healthy subjects.

Two hundred fourteen healthy subjects were also screened for the novel *CFHR4* CNVs using multiplex polymerase chain reaction (mPCR) that amplified intron 1 and exon 2 of *CFHR4* and intron 3 of *CFHR1* (Moore et al., 2010).

### Single Molecule Real-Time (SMRT) Sequencing

Probes targeting *CFH-CFHRs* on the human genome reference hg19 (from chr1:196619000 to chr1:196979303) were designed using online Nimble Design Software (Roche Sequencing, Pleasanton, CA, United States). Samples from 10 patients (new or rare SVs, *n* = 6; heterozygous *CFHR3-CFHR1* del, *n* = 1; homozygous *CFHR3-CFHR1* del, *n* = 1; heterozygous *CFHR1-CFHR4* del, *n* = 1; *CFHR3-CFHR1* del and *CFHR1-CFHR4* del compound heterozygote, *n* = 1) and 7 healthy controls (normal copy number, *n* = 4; heterozygous *CFHR3-CFHR1* del, *n* = 3) were sequenced at the Norwegian Sequencing Centre^1^. Patient #1678, in whom the boundaries of the *CFHR3_1__–__5_-CFHR4_10_* fusion gene had been previously characterized by Sanger sequencing was included as a positive control. Libraries were prepared using the Pacific Biosciences (PacBio) protocol for Target Sequence Capture using SeqCap^®^ EZ Libraries with PacBio^®^ Barcoded Adapters. Briefly, 2 μg of DNA were sheared to 7 kb. Amplified and barcoded DNA were size selected using BluePippin. After pooling, the template was hybridized using *CFH-CFHR* probes. Following amplification, libraries were size selected by BluePippin with a 5 kb cut-off and then sequenced using Pacbio Sequel system.

Data were obtained as multiplexed subreads and were demultiplexed with the PacBio read demultiplexer *lima*, retaining only those subreads with a barcode quality greater than 45. To ensure high quality sequencing data, we used PacBio Circular Consensus Sequencing (CCS, also known as HiFi) reads, produced by obtaining a consensus sequence from subreads. The CCS reads were obtained with a PacBio tool called *ccs* with the following parameters: *–minLength* = *1000*, *–min-rq* = *0.99* and *–maxLength* = *10,000*. The length of the resulting CCS reads ranged from 1,351 to 10,108 bp, and the number of sequencing passes ranged from 3 to 114. CCS reads were mapped to hg19 with two long-read mappers: NGMLR (with*-min-identity* = *0.95*) and *minimap2* (using pre-set CCS). SV calling was carried out with *Sniffles* for NGMLR-aligned reads and with *pbsv* for both aligners. While the results for patient #1678 matched those previously obtained by MLPA and Sanger sequencing (positive control), some SVs involved large repeated regions and were difficult to resolve.

As shown in Supplementary Figure 1, the target region is characterized by two intralocus large SDs and a number of shorter repeats. The first duplicated region (b1 and b2, blue in Supplementary Figure 1) is 28,650 bp long (b1) and has an identity of around 98% with its counterpart (b2), which is 28,726 bp long. The second duplicated region (r1 and r2, red in Supplementary Figure 1) is 40,218 bp long (r1) and has an identity of ∼97% to its 39,726 bp long counterpart (r2). The *CFHR3-CFHR1* del CNV occurs across the b1/b2 duplications, while the *CFHR1-CFHR4* del occurs across the r1/r2 duplications. These regions are much longer than our average CCS read length (∼6,000 bp) and therefore while the “signature” of SVs involving these repeated regions typically could be detected by inspecting alignments (for example, as split-read alignments) or by reviewing the SV caller output, similar “signatures” were also observed in non-carriers (false positives). Supplementary Figure 2 shows 3 individuals, *CFHR1-CFHR4* del, *CFHR3-CFHR1* del, normal control, who all show split-read alignments across the duplicated regions in spite of different genotypes. This example of a false positive likely reflects mapping errors caused by fragments originating in one region but mapping to the paralogous region, thereby generating a pattern similar to that associated with true SVs. We were, however, able to identify and locate SV breakpoints outside the repeated regions (see “Results” section) either by inspecting the aligned reads with Integrative Genomics Viewer (IGV) or based on the SV callers.

### Western Blot

The molecular pattern of FH-FHRs was studied by Western Blot (WB) using serum/plasma (diluted 1:40 for FHRs and 1:80 for FH). Proteins were separated by 10–12% SDS-PAGE (Mini-Protean TGX Precast Gels, Bio-Rad) under non-reducing conditions and transferred by electroblotting to polyvinylidene Difluoride (PVDF) membrane (*Trans*-Blot® Turbo^TM^ Midi PVDF Transfer; Bio-Rad). Membranes were blocked in 5% fat free (skim) milk and developed using specific FH/FHR antibodies: the FHR3 polyclonal antiserum and the monoclonal anti-FHR1 antibody (JHD) were a kind gift from Prof. Zipfel (Skerka et al., 2013) while the anti-FHR1-2-5 monoclonal antibody was kindly provided by Prof. de Cordoba (Goicoechea de Jorge et al., 2013). Factor H was detected using the commercial monoclonal anti-human Factor H (OX-23, LSBio). Incubation with primary antibodies was followed by horseradish peroxidase (HRP) conjugated secondary antibodies and ECL chemiluminescence detection system (Amersham).

### Statistical Analysis

Chi-square or Fisher’s exact tests were used to analyze categorical variables, while ANOVA was used to test continuous variables. Correction for multiple tests was applied.

## Results

### Patients

One hundred ninety-nine patients with primary C3G or IC-MPGN were recruited from the Italian Registry of MPGN (IC-MPGN: *n* = 96, 48.2%; C3G: *n* = 103, 51.8%, including C3GN: *n* = 74; DDD: *n* = 29), 159 of whom have been described in a previous study (Iatropoulos et al., 2018). The mean age at diagnosis was 18.6 ± 14.9 years (range: 0.3–72 years; IC-MPGN: 19.9 ± 15.1 years; C3GN: 18.3 ± 16 years; DDD: 15.2 ± 10.5 years).

Using the published three-step algorithm (Iatropoulos et al., 2018), patients were assigned to clusters 1 (*n* = 66), 2 (*n* = 49) and 3 (*n* = 33) or cluster 4 (*n* = 51) (Table 1). As expected, plasma sC5b-9 levels were significantly higher in clusters 1 and 2 than in cluster 3.

**TABLE 1:** Histologic diagnosis, complement assessment and genetic screening of patients recruited selected from the Italian Registry of MPGN and classified into clusters using the three-step algorithm.

	Cluster 1 (*n* = 66)	Cluster 2 (*n* = 49)	Cluster 3 (*n* = 33)	Cluster 4 (*n* = 51)	Overall *P-*value
IC-MPGN (*n* = 96)	21.2%	89.8%	12.1%	66.7%	<0.0001
C3GN (*n* = 74)	78.8%	10.2%	0%	33.3%	<0.0001
DDD (*n* = 29)	0%	0%	87.9%	0%	<0.0001
Sex,% men	57.6%	49%	63.6%	60.8%	0.54
Age (yr)-Mean (*SD*)	14 (±10.7)	18.6 (±13.7)	15.3 (±10.2)	26.9 (±19.5)^a,b,c^	<0.001
Serum C3 (mg/dl)	29.1 (±19.8)	22.8 (±21.9)	35.4 (±34.3)	93.2 (±26.1)^a,b,c^	<0.001
Serum C4 (mg/dl)	18.7 (±6.7)	16.8 (±11.3)	20.6 (±8.7)	21 (±10)	0.11
Plasma sC5b-9 (ng/ml)	1378 (±1255)^c,d^	1861 (±1357)^c,d^	540 (±604)^a,b^	302 (±145)^a,b^	<0.001
Low serum C3	100%	100%	93.9%	49%^a,b,c^	<0.001
Low serum C4	7.7%	28.6%^a,c,d^	6.2%	10%	0.004
Low serum C3 and normal C4	92.3%^b^	71.4%^a^	87.5%	44%^a,c^	<0.001
High plasma sC5b-9	76.3%	83%	32.3%^*a,b*^	20%^a,b^	<0.001
RV carriers	27.3%	22.4%	15.2%	3.9%^a,b^	0.01
C3NeF positive	53.4%	62%	79.3%^*a*^	5.9%^a,b,c^	<0.001
RV carriers and/or C3NeF	71.6%	74.5%	82.8%	9.8%^a,b,c^	<0.001
FH levels (mg/L)	301.9 (±70.9)	284.5 (±72)	275.7 (±65)	322.5 (±76)^b,c^	0.02
Low FH levels	6.9%	8.7%	6.9%	0%	0.26
Anti-FH antibodies	1.7%	6.5%	10.3%	0%	0.08

*Quantitative variables are expressed as mean (±SD). Abbreviations and limit of normal range: C3: 90–180 mg/dl. C4: 10–40 mg/dl; Normal plasma sC5b-9 levels: ≤400 ng/ml; Normal serum/plasma FH levels: ≥193 mg/L; RV, rare variant defined as genetic variant in coding and splicing regions of complement genes already related to C3G- IC-MPGN (CFH, CFI, CD46, CFB, C3, and THBD) with minor allele frequency (MAF) in the gnomAD database <0.001 and with Combined Annotation Dependent Depletion (CADD) phred score ≥10; p-values were corrected for multiple tests. ^*a*^Significant different from cluster 1; ^*b*^significant different from cluster 2; ^*c*^significant different from cluster 3; ^*d*^significant different from cluster 4.*

### Serum Factor H Abnormalities

Circulating factor H (FH) levels were measured in 181 patients and were lower than normal (reference ≥193 mg/L) in 9 patients (5%; C3G, *n* = 5; IC-MPGN, *n* = 4), all from clusters 1, 2, and 3. Six of the nine patients carried *CFH* RVs (N-terminal RV, *n* = 5; SCR15 -mid-region of FH-, *n* = 1) compared to 4 of 172 patients with normal FH levels (Table 2).

**TABLE 2:** List of patients with low FH levels and/or genetic or acquired FH abnormalities.

Pat ID	Histol. group	Algor. cluster	Age of onset (y)	Rare variant	Zyg.	gnomAD global freq.	CADD	Variant classif.	SVs	C3NeF	Serum C3 (mg/dl)	Serum C4 (mg/dl)	Plasma sC5b-9 (ng/ml)	FH levels (mg/dl)	FHAAs
1073	IC	2	28	p.FH: C494R^a^ (SCR8)	Het	0	24.0	LPV*	Normal	Neg	70	28	1332	180	Neg
1304	IC	2	30.6	No	/	/	/	/	Heterozygous *CFHR3-CFHR1* del	Pos	45	9	249	91	Neg
1773	DDD	3	11.8	No	/	/	/	/	Normal	Pos	9	24	267	91	Neg
2032	C3GN	1	24	p.FH: R78G^a,b,c,d^ (SCR1)	Hom	0	16	P	Normal	Neg	15	27.5	1530	133	Neg
2082	C3GN	1	0.75	p.FH: R127C^a, b^ (SCR2)	Het	0	33	LPV	Normal	NA	72	26	2789	151	Neg
2158	C3GN	1	41.7	p.FH: R78G^a,b,c,d^ (SCR1)	Hom	0	16	P	Normal	Neg	14	35	4571	154	Neg
2192	C3GN	1	9	p.FH: G133R^a^ (SCR2)	Het	8E-06	31	LPV^§^	Normal	Neg	55	20	355	178	Neg
2585	IC	2	8.8	p.FH: G879R^b^ (SCR15)	Het	4E-06	28	LPV	Normal	Pos	49	5	1209	119.5	Neg
2888	IC	2	16	No	/	/	/	/	Heterozygous *CFH-CFHR3-CFHR1* del	Neg	8.5	23	253	156	Neg
1026	IC	2	7	No	/	/	/	/	Normal	Pos	5	6	1080	222	Pos
1837	DDD	3	10.6	No	/	/	/	/	Homozygous *CFHR3-CFHR1* del	Pos	9	29	545	264	Pos
1967	IC	3	22	No	/	/	/	/	Heterozygous *CFHR3-CFHR1* del	Pos	84	28.4	257	265	Pos
2047	IC	2	10	p.C3: S1063N^a,b^ (TED domain)	Het	6.9E-05	10	VUS	Normal	Neg	70	6	235	315	Pos
2081	IC	2	8.5	No	/	/	/	/	Normal	Pos	16	13.1	1643	313	Pos
2163	IC	3	6.5	No	/	/	/	/	Normal	Pos	18	19	277	376	Pos
2557	IC	1	4.8	No	/	/	/	/	Normal	Neg	33	11	605	267	Pos
1101	DDD	3	48.7	p.FH: R1210C^a,b,e,f^ (SCR20)	Het	1.5E-04	12	P	Normal	Neg	154	14	368	394	Neg
1284	IC	2	0.4	p.FH: P88T^a,b^ (SCR2)	Hom	0	29	LPV	Normal	Neg	5.4	24.7	3596	NA*	NA
1287	IC	2	0.3	p.FH: P88T^a,b^ (SCR2)	Hom	0	29	LPV	Normal	Neg	47.7	45.3	2074	NA*	NA
1549	DDD	3	24.7	p.FH: R2I^a^ (Signal peptide)	Het	0	11	VUS	1 copy of *CFHR3* + 3 copies of *CFHR4*	Pos	54	18	286	216	Neg

*FH abnormalities are highlighted with gray color. Abbreviations and limit of normal range: Pat. ID, patient ID; Histol. Group, histologic group according to the current classification; Algor. Cluster, cluster group assigned using three-step algorithm; y, years; Zyg, zygosity; Rare variant is defined as genetic variant in coding and splicing regions of complement genes with minor allele frequency (MAF) in the gnomAD (genome aggregation database) <0.001 and with CADD (Combined Annotation Dependent Depletion) phred score ≥10. gnomAD Freq., MAF in all subjects of the gnomAD database (v2.1.1); Variant Classif., variant Classification reported in the Database on complement gene variant (https://www.complement-db.org/home.php) based on guidelines from ACMG (Richards et al., 2015) and from the KDIGO conference on aHUS and C3G (Goodship et al., 2017). When variant classification was not available in the database, the variant was classified using in silico predictions on the basis of KDIGO guidelines. P, pathogenic; LPV, likely pathogenic variant; VUS, variant of uncertain significance. SVs, structural variants defined as genomic rearrangements longer than 1 kb.*

*C3NeF, C3 nephritic factor;*

*C3, 90–180 mg/dl;*

*C4, 10–40 mg/dl;*

*Normal plasma sC5b-9 levels: ≤400 ng/ml;*

*Normal serum/plasma FH levels: ≥193 mg/L;*

*FHAAs, anti-FH antibodies.*

*^*a*^Iatropoulos et al. (2018).*

*^*b*^Osborne et al. (2018).*

*^*c*^Pechtl et al. (2011).*

*^*d*^Caprioli et al. (2003).*

*^*e*^Servais et al. (2012).*

^*f*^Maga et al. (2010).

*Pathogenic in 11 of 11 in silico tools;

*^§^Pathogenic in 10 of 11 in silico tools.*

Screening for FH autoantibodies (FHAAs) identified 7 (4%) positive patients, all from clusters 1, 2, and 3 (IC-MPGN, *n* = 6; C3G, *n* = 1) (Table 2). All 7 patients had low C3 and normal FH levels, although 2 had low C4 levels. In addition to FHAAs, 5 patients were co-positive for C3NeFs. One patient negative for C3NeFs carried a RV in *C3* (p.Ser1063Asn; gnomAD global MAF = 6.9 × 10^–5^). Six of the seven patients experienced childhood disease onset (ranging from 4.8 to 10.6 years).

### *CFH-CFHR* Copy Number Variations

**FIGURE 5:**
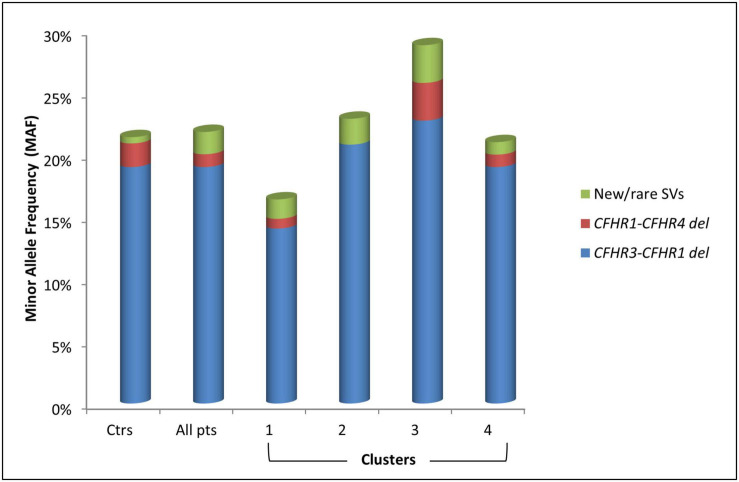
Histogram representing the Minor Allele Frequency (MAF) and distribution of common (*CFHR3-CFHR1* del and *CFHR1-CFHR4* del) and new/rare structural variants (SVs) in patients (pts) and in healthy controls (ctrs).

Common CNVs, namely the *CFHR3-CFHR1* (*CFHR3-CFHR1* del) and/or the *CFHR1-CFHR4* (*CFHR1-CFHR4* del) deletions, were identified in 32.8% of patients and 36.9% of controls (Figure 5 and Table 3). Although there was no difference in the prevalence of the homozygous *CFHR3-CFHR1* del when patients and controls were compared, across patient groups, the homozygous *CFHR3-CFHR1* del was more frequently observed in cluster 3 than in cluster 1 (Table 3). This relationship remained when we also included two patients (one in cluster 1 and one in cluster 3) who were compound heterozygotes for *CFHR3-CFHR1* del and *CFHR1-CFHR4* del. There was no association between the homozygous *CFHR3-CFHR1* del and FHAAs.

**TABLE 3:** Frequency of common structural variants (SVs) in patients and in controls.

Common SVs	Ctrs	All patients (*n* = 195)			Cluster 1 (*n* = 64)			Cluster 2 (*n* = 48)			Cluster 3 (*n* = 33)			Cluster 4 (*n* = 50)		
		Freq (*n*)	OR (95% Cl)	*P-*value^a^	Freq (*n*)	OR (95% Cl)	*P-*value^a^	Freq (*n*)	OR (95% Cl)	*P-*value^a^	Freq (*n*)	OR (95% Cl)	*P-*value^a^	Freq (*n*)	OR (95% Cl)	*P-*value^a^
Het*CFHR3-CFHR1*del	32% (32/100)	27.7% (54)	0.81 (0.5–1.4)	0.44	26.6% (17)	0.77 (0.4–1.5)	0.46	31.2% (15)	0.97 (0.46–2)	0.93	18.2% (6)	0.47 (0.2–1.3)	0.13	32% (16)	1 (0.5–2.2)	1
Hom*CFHR3-CFHR1*del	3% (3/100)	3.1% (6)	1.03 (0.2–4.2)	0.97	0	0.22 (0–4.2)	0.31	4.2% (2)	1.41 (0.2–8.7)	0.71	9.1% (3)	2.7 (0.6–16.9)	0.16	2% (1)	0.85 (0.1–6.5)	0.72
*CFHR3-CFHR1*del+ *CFHR1-CFHR4*del	0% (0/214)	1% (2)	5.54 (0.3–116.2)	0.27	1.6% (1)	10.14 (0.4–251.8)	0.16	0	4.42 (0.1–225.7)	0.46	3% (1)	19.8 (0.8–496.5)	0.07	0	4.25 (0.1–216.6)	0.47
Het*CFHR1-CFHR4*del	1.9% (4/214)	1% (2)	1.84 (0.3–10.2)	0.48	0	2.75 (0.2–51.9)	0.5	0	2.07 (0.1–39.2)	0.62	3% (1)	0.61 (0.1–5.6)	0.66	2% (1)	0.93 (0.1–8.5)	0.95

*^*a*^P-values were calculated vs. controls (ctrs). Patients carrying both rare/new and common SVs were excluded.*

Seven patients (3.6%) carried novel or rare CNVs that included a hybrid gene, two gene deletions, and four gene duplications. The new or rare CNVs were distributed among all clusters (Figure 5). Histologic, biochemical and genetic data of these patients are reported in Table 4.

**TABLE 4:** List of patients carrying new/rare SVs.

Family ID	Patient ID	Gender	Histologic diagnosis	Algorithm-based cluster	Age of onset (years)	New/rare SVs	Serum C3 levels (mg/dl)	Serum C4 levels (mg/dl)	Plasma sC5b-9 levels (ng/ml)	RV	C3NeF	FHAAs	FH levels (mg/dl)
913	1678	Female	DDD	3	26	*CFHR3-CFHR4* hybrid gene + *CFHR3-CFHR1* del	72.5	20	269	No	Neg	Neg	323
1876	2870	Female	C3GN	4	50	*partial CFHR3 deletion* + *CFHR3-CFHR1* del	94.7	65.3	470	No	Neg	Neg	573
1892	2888	Male	IC	2	16	*CFH-CFHR3-CFHR1* deletion	8.5	23	253	No	Neg	Neg	156
1866	2856	Male	C3GN	1	11	3 copies of *CFHR1* + 3 copies of *CFHR4*	9	17.1	1930	No	Pos	Neg	454
1970	2979	Male	C3GN	1	5	3 copies of *CFHR1* + 3 copies of *CFHR4*	51	19	NA	No	NA	NA	NA
950	1726	Female	IC	2	25	1 copy of *CFHR3* + 3 copies of *CFHR4*	20	6	291	*CFB:* p. R679W	Neg	Neg	393.5
811	1549	Female	DDD	3	25	1 copy of *CFHR3* + 3 copies of *CFHR4*	54	18	286	*CFH:* p.R2I	Pos	Neg	216

*Abbreviations and limit of normal range: SVs, structural variants defined as genomic rearrangements resulting in duplications, deletions and inversions larger than 1 kb; NA, not available; C3, 90–180 mg/dl; C4, 10–40 mg/dl; Normal plasma sC5b-9 levels: ≤400 ng/ml; Normal serum/plasma FH levels: ≥193 mg/L; RV, rare variant defined as genetic variant in coding and splicing regions of complement genes already related to C3G- IC-MPGN (CFH, CFI, CD46, CFB, C3, and THBD) with MAF in the gnomAD database <0.001 and with Combined Annotation Dependent Depletion (CADD) phred score ≥10; C3NeF, C3 nephritic factor; FHAAs, anti-FH antibodies.*

#### *CFHR3_1__–__5_-CFHR4_10_* Hybrid Gene

**FIGURE 6:**
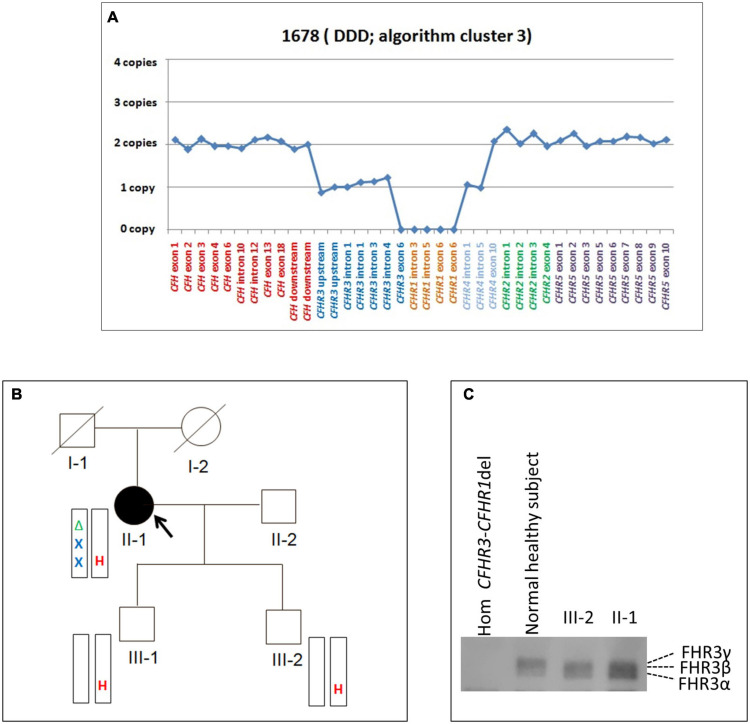
The *CFHR3_1__–__5_-CFHR4_10_* hybrid gene identified in a DDD patient in cluster 3. **(A)** Results of MLPA showing in patient #1678 two normal copies of *CFH*, only one copy of *CFHR3* lacking exon 6, zero copies of *CFHR1*, one normal and one partially deleted copy of *CFHR4* and two copies of *CFHR5*. **(B)** Pedigree (#913) of the DDD patient (II-1; indicated by the black circle) carrying the *CFHR3_1__–__5_-CFHR4_10_* hybrid gene on one allele and the *CFHR3-CFHR1* del on the other allele. The *CFHR3_1__–__5_-CFHR4_10_* hybrid gene is indicated in red (H) and the *CFHR3-CFHR1* del is indicated in green (Δ). The patient also carries two heterozygous nonsense rare variants of unknown significance (VUS) in the *CFHR2* (p.Gln211Ter - rs41299605 – and p.Arg254Ter – rs41313888 -; gnomAD global MAF: 6.5 × 10^–5^ and 7.5 × 10^–4^, respectively), indicated in blue (X). The *CFHR3_1__–__5_-CFHR4_10_* hybrid gene, but not the *CFHR2* rare variants (RVs) and the *CFHR3-CFHR1* del, was transmitted to the two healthy patients’ sons (III-1 and III-2). **(C)** Western Blot (WB) of FHR3 was performed using an anti-FHR3 polyclonal antiserum (diluted 1:2,000), under non-reducing conditions, using the sera from the proband (II-1), her healthy son (III-2), a healthy control with normal CNVs (positive control) and a patient carrying the homozygous *CFHR3-CFHR1* del (negative control). The presence of 3 bands in the proband, corresponding to the different glycosylated variants of FHR3, indicates that the FHR3_1__–__4_-FHR4_9_ hybrid protein is secreted, since she is *CFHR3-CFHR1* deleted on the other allele.

A new deletion involving *CFHR3*, *CFHR1* and *CFHR4* genes was identified in 1 patient (cluster 3; DDD; Patient #1678; Table 4) who presented with proteinuria (2 g/day) and low C3 levels (C3 = 45 mg/dl) at the age of 26. Her renal impairment progressed from the age of 32, reaching end-stage renal disease (ESRD) by age 38. She has received 3 kidney transplants, losing the first and second allografts to disease recurrence. Prior to her third transplant, she had slightly reduced C3 (72.5 mg/dl) but normal C4 (23 mg/dl), sC5b-9 (269 ng/ml), and FH (323 mg/L) levels. C3NeFs and FHAAs were absent and genetic screening failed to identify any RVs in *CFH*, *C3*, *CD46*, *CFI*, *CFB*, and *THBD*. CNV analysis was remarkable for one copy of *CFHR3* that lacked exon 6, zero copies of *CFHR1*, and two copies of *CFHR4*, one of which carried a large deletion (Figure 6A). Long PCR and Sanger sequencing confirmed a deletion extending from exon 6 of *CFHR3* to exon 9 of *CFHR4*, predicting a novel *CFHR3_1__–__5_-CFHR4_10_* hybrid gene. The breakpoint region was mapped between chr1:196760556 (intron 5 of *CFHR3*) and chr1:196886396 (intron 9 of *CFHR4*). Within the breakpoint region, we identified an insertion of 305 bp with sequence similarity to the two Alu Repeats located in intron 5 of *CFHR3* and in intron 9 of *CFHR4* (Supplementary Figure 3).

**FIGURE 7:**
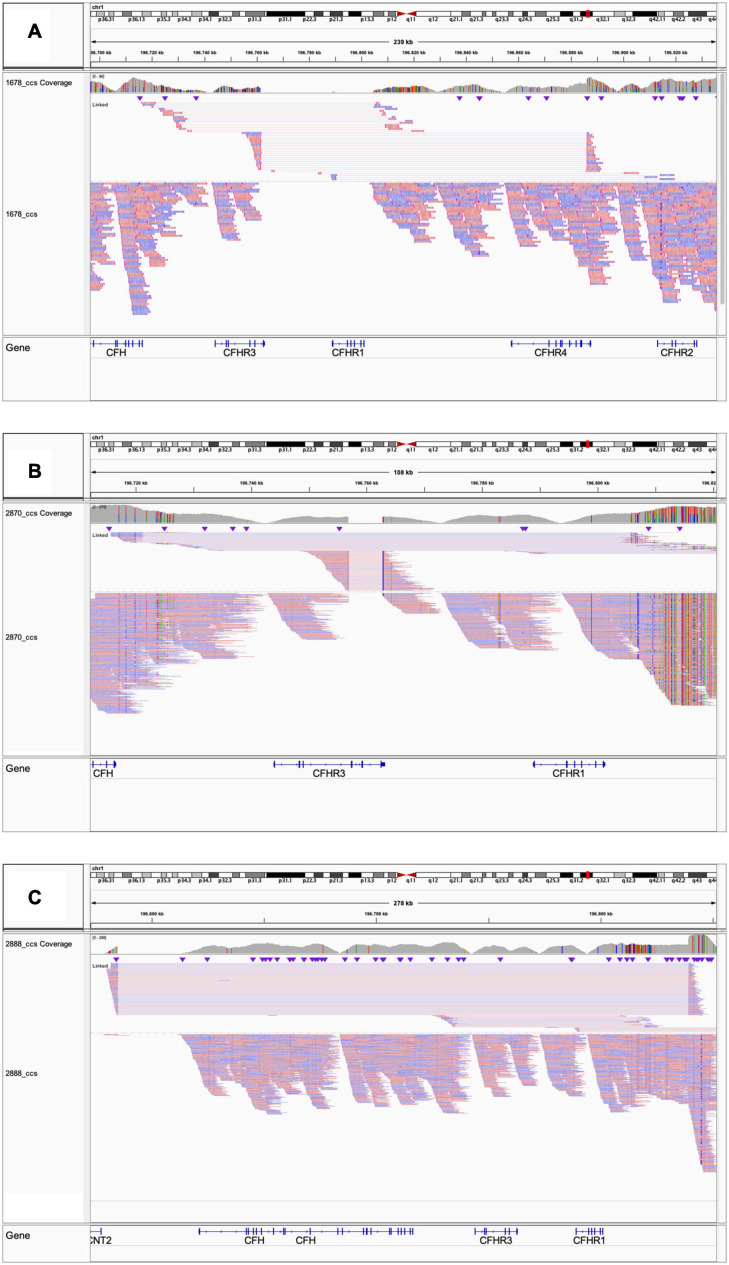
Screenshot from IGV (Integrative Genomics Viewer) showing reads from SMRT sequencing. **(A)** Patient #1678 carrying the *CFHR3_1__–__5_-CFHR4_10_* and the *CFHR3-CFHR1* del. **(B)** Patient #2870 carrying the *CFHR3*-intron 3 to *CFHR3*-3′UTR deletion and the *CFHR3-CFHR1* del. **(C)** Patient #2888 carrying the heterozygous deletion of *CFH*, *CFHR3*, and *CFHR1.*

Because the *CFH-CFHR1-5* genomic region has several duplicated regions and a large number of Alu repeats that represent a strong limitation for sequence characterization of *CFH-CFHR* genomic rearrangements, we used SMRT, a DNA sequencing long-read approach. SMRT correctly identified the *CFHR3_1__–__5_-CFHR4*_10_ hybrid gene on one allele and distinguished it from the *CFHR3-CFHR1* del present on the other allele in the positive control (patient #1678 DNA), and confirmed the breakpoint region identified by Sanger sequencing (Figure 7A).

It is noteworthy that patient #1678, who belongs to cluster 3, is completely deficient in *CFHR1*.

The same *CFHR3_1__–__5_-CFHR4_10_* hybrid gene was detected in this patient’s two unaffected sons (Figure 6B) and in one of 214 healthy controls. WB using a polyclonal anti-human FHR3 antibody showed that the *CFHR3_1__–__5_-CFHR4_10_* hybrid gene generates a FHR3_1__–__4_-FHR4_9_ hybrid protein (II-1; Figure 6C).

To search for additional genetic abnormalities in *CFHR* genes that may contribute to the disease phenotype in the patient, we performed targeted sequencing using CasCADE and identified two heterozygous nonsense RVs on the same allele in *CFHR2* (p.Gln211Ter – rs41299605 – and p.Arg254Ter – rs41313888 -; gnomAD global MAF: 6.5 × 10^–5^ and 7.5 × 10^–4^, respectively) that were not transmitted to her healthy sons (Figure 6B).

#### *CFHR3* Deletion

**FIGURE 8:**
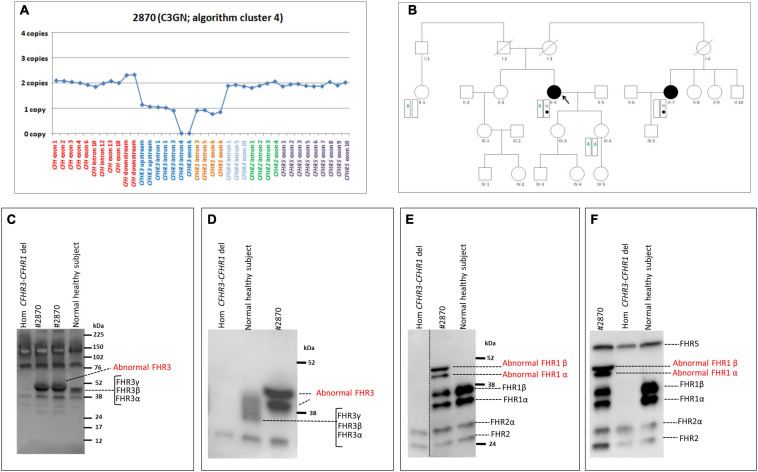
The *CFHR3* deletion identified in the C3GN patient in cluster 4. **(A)** Results of MLPA showing two normal copies of *CFH*, one copy of *CFHR3* until intron 3, zero copies of *CFHR3* from intron 4 to exon 6, one copy of *CFHR1* and two normal copies of *CFHR4*, *CFHR2* and *CFHR5*. **(B)** Pedigree (#1876) of the C3GN patient (II-4; indicated by the black circle) carrying the *CFHR3* SV (H, indicated in red) on one allele and the *CFHR3-CFHR1* del (Δ, indicated in green) on the other allele. The patient also carries a variant of unknown significance (VUS; indicated with a filled circle) in *CFHR4* (p.Val438Gly; rs766466004; gnomAD global MAF: 4 × 10^–6^). Both the *CFHR3* SV and the *CFHR4* VUS were also found in the maternal cousin (II-7, indicated by the black circle) who has an MPGN diagnosis but, not in the patient’s healthy sons. **(C–F)** Western Blot (WB) analyses were performed under non-reducing conditions using the sera from the proband (#2870), a healthy control with normal CNVs (positive control) and a patient carrying the homozygous *CFHR3-CFHR1* del (negative control). Using the rabbit anti-FHR3 polyclonal antiserum (diluted 1:2000; panel **C**,**D**) we did not observe the predicted band of the shorter FHR3 at 16 kDa (**C**; predicted MW based on the partial *CFHR3* deletion). Instead we observed two bands with a MW (around 50 kDa) higher than normal FHR3, which are better evidenced in **(D)**, obtained after a longer run. Using an anti-FHR1 antibody (JHD; diluted 1:1,000) we found both the two bands corresponding to normal glycosylated isoforms of FHR1 (around 37 and 41 kDa, respectively) and two abnormal bands around 50 kDa, identical to those observed with the anti-FHR3 antiserum **(E)**. The same WB pattern was confirmed using the anti-FHR1-2-5 antibody (2C6; **F**). Altogether the WB findings indicate the presence in the proband of both the normal FHR1 and a fusion protein encompassing FHR3 and FHR1.

In a patient from cluster 4 (#2870; Table 4), MLPA revealed 1 copy of *CFHR3* to intron 3, 0 copies of *CFHR3* from intron 4 to exon 6, and 1 copy of *CFHR1* (Figure 8A). The patient, who had a family history of nephropathy, developed disease heralded by microhaematuria and proteinuria at 50 years of age. Because proteinuria persisted (0.6–1.0 g/day for at least 8 years), at age 58, a kidney biopsy was performed and a diagnosis of C3GN was made. Serum protein electrophoresis was normal and the patient was negative for C3NeFs and FHAAs. Six years later, proteinuria increased to the nephrotic-range (3.7 g/day), renal function declined (creatinine 1.3 mg/dl), and treatment with diuretics, angiotensin-converting-enzyme (ACE) inhibitors and angiotensin II receptor blockers (ARBs) was initiated. At last follow-up, creatinine was 1.1 mg/dl, C3 (94.7 mg/dl) and C4 (65.3 mg/dl) were normal, and sC5b-9 was slightly increased (470 ng/ml).

We were not able to identify the deletion breakpoints by long PCR and Sanger sequencing; however, SMRT sequencing showed that the abnormal MLPA pattern derived from both the *CFHR3-CFHR1* del on one allele and a novel deletion from *CFHR3*-intron 3 to *CFHR3*-3′UTR on the other allele (Figure 7B). SMRT data also identified the two genomic breakpoints (hg19: chr1:196756789 at *CFHR3* intron 3 and chr1:196762816 at *CFHR3* 3′UTR), which were confirmed by long PCR and Sanger sequencing using primers targeting the breakpoint region (Supplementary Table 2). These data indicate the presence of a shorter *CFHR3* gene comprised of only exons 1, 2, and 3. In addition, NGS identified a heterozygous RV in *CFHR4* (p.Val438Gly; rs766466004; gnomAD global MAF: 4 × 10^–6^; II-4, Figure 8B). Both the partial *CFHR3* deletion and the *CFHR4* rare variant were identified in a maternal female cousin (II-7; Figure 8B) with a history of proteinuria from the age of 15 and a biopsy diagnosis of MPGN (IF and EM data are not available). She developed progressive chronic renal failure and received a kidney transplantation 33 years after onset. Neither the *CFHR3* genomic abnormality nor the *CFHR4* variant were identified in the unaffected patient’s daughter (III-4; Figure 8B) or in a healthy paternal female cousin (II-1; Figure 8C,D) or in 100 healthy controls.

The predicted MW of the protein encoded by the partially deleted *CFHR3* gene is about 16 kDa. However, WB analyses of patient serum using an anti-FHR3 antibody showed two bands with a MW around 50 kDa and no bands at 16 kDa (Figures 8C,D). Western blot with an anti-FHR1 antibody revealed two bands corresponding to normal glycosylated isoforms of FHR1 and two additional bands with MWs (about 50 kDa; Figure 8E) identical to the bands observed with the anti-FHR3 antibody. The same results were observed with an anti-FHR1-2-5 antibody (Figure 8F). These results suggest the presence of 1) a hybrid protein between the shorter FHR3 and the full FHR1 (likely FHR3_1__–__3_-FHR1); 2) a normal FHR1.

#### *CFH-CFHR3-CFHR1* Gene Deletion

**FIGURE 9:**
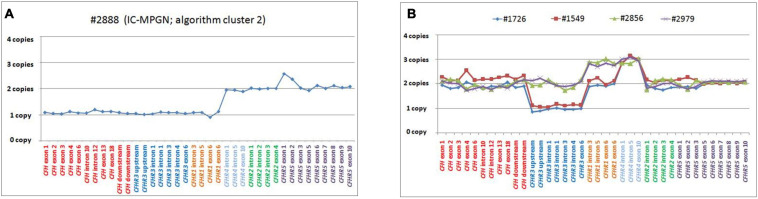
Graphic representation of MLPA results from the patient carrying the *CFH-CFHR3-CFHR1* deletion and patients with *CFHR4* duplication. **(A)** MLPA results showing the genomic deletion, including the entire copy of *CFH*, *CFHR3*, and *CFHR1* in a patient with IC-MPGN (#2888; cluster 2). **(B)** Analysis of MLPA showing three copies of *CFHR1* and *CFHR4* in two C3GN patients (#2856 and #2979; both from cluster 1) and one copy of *CFHR3*, two copies of *CFHR1* and 3 copies of *CFHR4* in two patients with IC-MPGN and DDD (#1726 cluster 2; #1549, cluster 3), respectively.

Heterozygosity for a large deletion that included *CFH*, *CFHR3* and *CFHR1* was identified by MLPA analysis (Figure 9A) in a patient in cluster 2 with histologic diagnosis of IC-MPGN (#2888; Table 4). At the age of 16, the patient presented with nephrotic syndrome, haematuria, low C3 levels (8.5 mg/dl), normal C4 and hypertension. No family history of nephropathy was reported. After 25 years, renal function deteriorated and the patient underwent a pre-emptive kidney transplantation (the donor was his father). Two years later, the patient lost the allograft due to rejection and started dialysis. At that time, biomarkers showed low C3 (47 mg/dl) and normal C4 (27 mg/dl). No C3NeF or FHAAs were detected and genetic screening did not reveal RVs in complement genes. Consistent with the deletion of one copy of *CFH*, FH levels were low (156 mg/dl). SMRT sequencing confirmed a 254 kb long deletion from chr1:196584749 (between *KCNT2* and *CFH*) and extending to chr1:196839345 (in the *CFHR1-CFHR4* intergenic region) (Figure 7C). Breakpoints were confirmed by Sanger sequencing (primers are reported in Supplementary Table 2). This deletion was not identified in any controls.

#### Gene Duplications

Two young male patients from cluster 1 (#2856, #2979; Table 4) carried a duplication of *CFHR1-CFHR4* and therefore had 3 copies of both *CFHR1* and *CFHR4* (Figure 9B).

The first, patient #2856, presented with proteinuria and haematuria at age 11 and had biopsy-confirmed C3GN. In the following years, he experienced progressive proteinuria, peaking at 11.8 g/day at the age of 25. C3 levels were low (9 mg/dl), sC5b-9 levels were high (1,930 ng/ml) and he was C3NeF positive. Mycophenolate mofetil (MMF; 2g/day) and prednisone (PDN; 1 mg/kg) were initiated, with an associated reduction in proteinuria (1.1 g/day) and at last follow-up (at 26 years of age), C3 levels had improved, sC5b-9 levels had normalized (328 ng/ml), and C3NeF was absent.

The second case, patient #2979, presented with proteinuria (0.26 g/day), haematuria and low C3 (51 mg/dl) at the age of 5; one year later, because of the persistence of proteinuria, he underwent a kidney biopsy, which showed C3GN. At last follow-up, one year later, proteinuria had increased (0.69 g/day), renal function was normal (creatinine 0.34 mg/dl), and C3 levels remained low (66 mg/dl).

One patient from cluster 2 (#1726) with IC-MPGN also carried 3 copies of *CFHR4* (but at variance with the first two cases, she had only two copies of *CFHR1* and one copy of *CFHR3;* Figure 9B). Disease developed during pregnancy when she presented at age 25 with proteinuria, microhaematuria, low C3 (20 mg/dl) and C4 (6 mg/dl) but normal renal function. Post-pregnancy treatment included chronic immunosuppression (corticosteroids, cyclophosphamide, MMF) and antihypertensive therapies (ACE inhibitors and ARBs), and at 45 years of age, C3 and C4 levels were normal and proteinuria and haematuria resolved. At last follow-up (at the age of 46), creatinine was 0.9 mg/dl, C3 and C4 were 140 mg/dl and 12 mg/dl, respectively, and morning urine spot was negative for microhaematuria and slightly positive for proteinuria (155 mg/g creatinine, normal values < 200 mg/g). C3NeFs and FHAAs were absent. Segregation analysis showed that the patient inherited an allele with zero copies of *CFHR3*, one copy of *CFHR1* and two copies of *CFHR4* from the unaffected father (allele A, Supplementary Figure 4). The other allele is normal. Of the two unaffected sons, one has inherited the maternal abnormal allele A and the paternal *CFHR3-CFHR1* deletion allele (Supplementary Figure 4). NGS also identified homozygosity for the *CFB* RV (p.Arg679Trp, gnomAD global MAF: 0), inherited from the consanguineous healthy parents. The patient’s sons are heterozygous for this variant (Supplementary Figure 4).

The same MLPA pattern seen in #1726 was also identified in a patient in cluster 3, who was diagnosed with DDD at 25 years of age when he developed nephrotic range proteinuria (6.2 g/day) in the face of low C3 levels (54 mg/dl) (#1549; Table 4 and Figure 9B). Renal function and blood pressure remained normal and conservative therapy with statins and ACE inhibitors was initiated, resulting in progressive reduction of proteinuria to below the nephrotic range. The patient was C3NeF positive. The patient has remained stable and at last follow-up (at the age of 34) had sub-nephrotic range proteinuria (1.8 g/24 h) and normal renal function (creatinine 0.55 mg/dl). C3 remained low (64 mg/dl) but sC5b-9 was normal (142 ng/ml) and C3NeFs had resolved. Segregation analysis showed that the abnormal allele (allele A) was maternally inherited. NGS studies identified a heterozygous RV in *CFH* (p.Arg2Ile; gnomAD global MAF: 0) that does not appear to impact FH levels (216 mg/dl); this variant was also maternally inherited.

Notably, we were not able to discriminate between carriers (#2856, #1726, and #1549) and non-carriers of the *CFHR1-CFHR4* duplication with CCS reads, likely due to the fact that the breakpoints of this SV are in the r1/r2 duplicated regions, which can lead to erroneous mapping (Supplementary Figure 5), as described in the Section “Materials and Methods.” No controls had more than 2 copies of *CFHR1* and/or *CFHR4.*

## Discussion

Here we performed a comprehensive analysis to characterize genetic and acquired FH-FHR abnormalities in a large cohort of 199 C3G/IC-MPGN patients, classified into four clusters, with the main focus on *CFH-CFHR* CNVs.

Low FH levels and genetic and acquired FH abnormalities were identified only in patients in clusters 1–3, which are characterized by fluid-phase complement activation. Specifically, 7% of cluster 1–3 patients had *CFH* RVs, consistent with our results in a smaller cohort (Iatropoulos et al., 2018). FHAAs were also found in 5% of cluster 1–3 patients, all with childhood onset. All but 1 FHAA-positive patient were diagnosed with IC-MPGN, suggesting a possible link between FHAAs and immune-complexes in the glomeruli. In addition, the majority of patients with FHAAs were co-positive for another autoantibody, C3NeF, consistent with other reports (Blanc et al., 2015). These findings suggest a cumulative or synergistic effect of FHAAs and C3NeF in inducing fluid-phase AP overactivation although the specific contribution of each autoantibody remains unclear.

At variance with aHUS patients, we did not observe a correlation between the presence of FHAAs and homozygosity for *CFHR1* del in C3G/IC-MPGN patients, consistent with previous data (Blanc et al., 2015; Valoti et al., 2019; Zhang et al., 2020). In addition, the prevalence of the common SVs (*CFHR3-CFHR1* del or *CFHR1-CFHR4* del) did not differ between patients and healthy controls, indicating that common SVs are not risk factors for C3G/IC-MPGN. However, the finding that total deficiency for *CFHR1* was more frequent in patients in cluster 3 compared to patients in cluster 1, may indicate that FHR1 deficiency plays a role in driving the disease phenotype characteristic of cluster 3 patients.

To date, with the exception of the fusion protein (FHR5_1_,_2_-FHR5) identified in Greek Cypriot patients with C3GN, rare SVs in the *CFH-CFHR* region have been described in only a few familial cases of C3G. They have not been implicated in IC-MPGN. This knowledge gap reflects, in part, the high degree of similarity within the *CFH-CFHR* region, which is a strong limitation in designing specific probes for copy number variation (CNV) analysis and leads to an incomplete investigation of this locus.

To optimize the CNV analysis in this region, we used available and custom MLPA probes to provide an overview of SVs, which we then further resolved through PacBio long-read sequencing (SMRT, Single- Molecule Real-Time, sequencing). Using this protocol, we identified rare *CFH-CFHR* SVs in patients with IC-MPGN and an overall prevalence of 4% of new and rare *CFH-CFHR* SVs in C3G/IC-MPGN patients.

We detected a duplication of *CFHR1-CFHR4* in 2% of patients distributed amongst clusters 1–3 but not in cluster 4, often in combination with other complement RVs and/or the common *CFHR3-CFHR1* del. This duplication has also been identified in patients with aHUS and AMD (Bu et al., 2014; Cantsilieris et al., 2018). We verified segregation in healthy relatives indicating that, alone, the *CFHR1-CFHR4* duplication is not sufficient to induce disease and that other risk factors are required to determine the ultimate phenotype.

**FIGURE 10:**
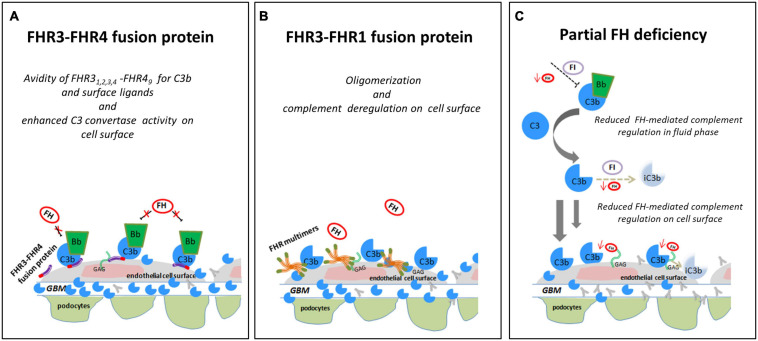
Hypothesis of the effects of FHR SVs on FH-complement regulation. **(A)** The hypothesis is that the FHR3_1−4_-FHR4_9_ fusion protein identified in a DDD patient (cluster 3) binds GAGs and C3b on glomerular cells, favouring the formation of an active AP C3 convertase that is resistant to FH-mediated decay, promoting the formation of highly electron-dense deposits in the glomerular basement membrane (GBM). **(B)** The FHR3-FHR1 fusion protein identified in a C3GN patient (cluster 4), through FHR1 portion, may generate multimeric complexes and through FHR3 domains increase the affinity of multimers for FH ligands and C3b, preventing FH-complement regulation (this process is known “FH deregulation”). The final effect is the bright C3 glomerular staining in the face of normal circulating C3. **(C)** In an IC-MPGN patient (cluster 2) we identified a heterozygous deletion of *CFH-CFHR3-CFHR1*. Low FH serum levels caused by the heterozygous deletion of the entire *CFH* gene may result in impaired FH-complement regulation both in the fluid phase and on the glomerular surface. The consequence is the deposition of C3b molecules on endothelial cells that promote glomerular chronic complement activation caused by immune-complexes.

Interestingly, another genomic rearrangement altering *CFHR4* was identified in a DDD patient from cluster 3, namely a *CFHR3_1__–__5_-CFHR4_10_* hybrid gene that encodes the fusion protein FHR3_1,2,3,4_-FHR4_9_. SCRs 1-3 of FHR3 have high sequence similarity with FH SCRs 6-8, which form a second FH heparan-sulfate binding site on cell surfaces and the glomerular basement membrane (GBM) (Borza, 2017). Hebecker and Jozsi have shown that FHR4 favors the assembly of the AP C3 convertase through its C-terminal region, which contains a C3b binding sites (Hebecker and Jozsi, 2012). These data suggest that the FHR3_1,2,3,4_-FHR4_9_ fusion protein may compete with FH for binding to both glycosaminoglycans/sialic acid and C3b fragments in the GBM, thereby enhancing C3 convertase activity and favoring the formation of the high electron-dense deposits, a characteristic feature of cluster 3-patients (Figure 10A). This hypothesis warrants testing.

In addition to the above *CFHR4* CNVs, in a familial case of C3GN in cluster 4 we identified a shorter *CFHR3_1__–__3_* gene caused by a deletion spanning intron 3 to 3′UTR, followed by a normal copy of *CFHR1*, which leads to a fusion protein likely consisting of the 2 N-terminal SCRs of FHR3 and the entire FHR1 (FHR3_1__–__2_-FHR1). A comparable fusion protein generated by a different genomic rearrangement has been described by Malik et al. (2012) in a familial C3GN case. In both cases, C3 levels are normal, suggesting that complement dysregulation occurs primarily in the glomeruli microenvironment. The likely mechanism of action is secondary to multimeric complexes of FH-related proteins that outcompete FH for binding to the glomerular glycomatrix (Goicoechea de Jorge et al., 2013; Medjeral-Thomas and Pickering, 2016; Csincsi et al., 2017) (Figure 10B). Functional studies, however, would be required to elucidate the functional effects of the identified genomic *CFHR* abnormalities and their pathogenetic role in C3G/IC-MPGN.

A final important finding of this study is the identification of a large deletion encompassing *CFH*, *CFHR3*, and *CFHR1* in a IC-MPGN patient in cluster 2 with low C3 and FH serum levels. The deletion causes FH haplodeficiency. As a consequence, fluid-phase AP regulation is impaired, which thereby sustains chronic complement activation initiated through the CP by immune-complexes in the glomeruli, a feature typical of cluster 2 patients (Figure 10C).

## Conclusion

In this study we have used established and innovative techniques to characterize SVs over the *CFH-CFHR* genomic region in a large cohort of C3G/IC-MPGN patients. We have demonstrated that while common *CFH-CFHR* SVs are not risk factors for disease, rare SVs do predispose to disease, but typically in combination with RVs in complement genes or acquired drivers of disease like autoantibodies. Our findings support the overarching concept that C3G/IC-MPGN are genetically complex, with the ultimate phenotype reflecting the delicate balance of serum levels of FH and the FHR proteins. Our results also illustrate the value of SMRT sequencing methodology as a tool for resolving the complexity of SVs in this genomic region.

## Data Availability Statement

The datasets generated for this study can be found in online repositories. The names of the repository/repositories and accession number(s) can be found below: EBI European Nucleotide Archive, accession no: PRJEB44176.

## Ethics Statement

The studies involving human participants were reviewed and approved by Ethics Committee of Bergamo. Written informed consent to participate in this study was provided by the participants’ legal guardian/next of kin. Written informed consent was obtained from the individual(s), and minor(s)’ legal guardian/next of kin, for the publication of any potentially identifiable images or data included in this article.

## Author Contributions

RP, MN, and GR designed research, interpreted data, and wrote the manuscript. RP, MB, MA, EV, PI, CM, RD, and PC performed the research and analyzed the data. EB provided detailed clinical information of patients. AB and RS analyzed the data and critically revised the manuscript. All authors contributed to the article and approved the submitted version.

## Conflict of Interest

MN has received honoraria from Alexion Pharmaceuticals for giving lectures, and for participating in advisory boards and research grants from Omeros, ChemoCentryx, Inception Science Canada, BioCryst Pharmaceuticals. AB has received honoraria from Boehringer Ingelheim, Alexion Phamaceuticals, Janssen Pharmaceuticals, Akebia Therapeutics, Inception Science Canada, BioCryst Pharmaceuticals. GR has consultancy agreements with Boehringer Ingelheim, Janssen Pharmaceuticals, Akebia Therapeutics, Alexion Pharmaceuticals, Alnylam, Inception Science Canada, BioCryst Pharmaceuticals. RS directs the Molecular Otolaryngology and Renal Research Laboratories, which provides genetic testing for complement-mediated renal diseases. None of these activities have had any influence on the results or interpretations in this article. The remaining authors declare that the research was conducted in the absence of any commercial or financial relationships that could be construed as a potential conflict of interest.

## Footnotes

1 www.sequencing.uio.no
