# Supplementary figures and images for: CFH and CFHR Copy Number Variations in C3 Glomerulopathy and Immune Complex-Mediated Membranoproliferative Glomerulonephritis
Source: Front Genet. 2021 Jun 11;12:670727. doi: 10.3389/fgene.2021.670727 (PMC8240960; doi:10.3389/fgene.2021.670727)

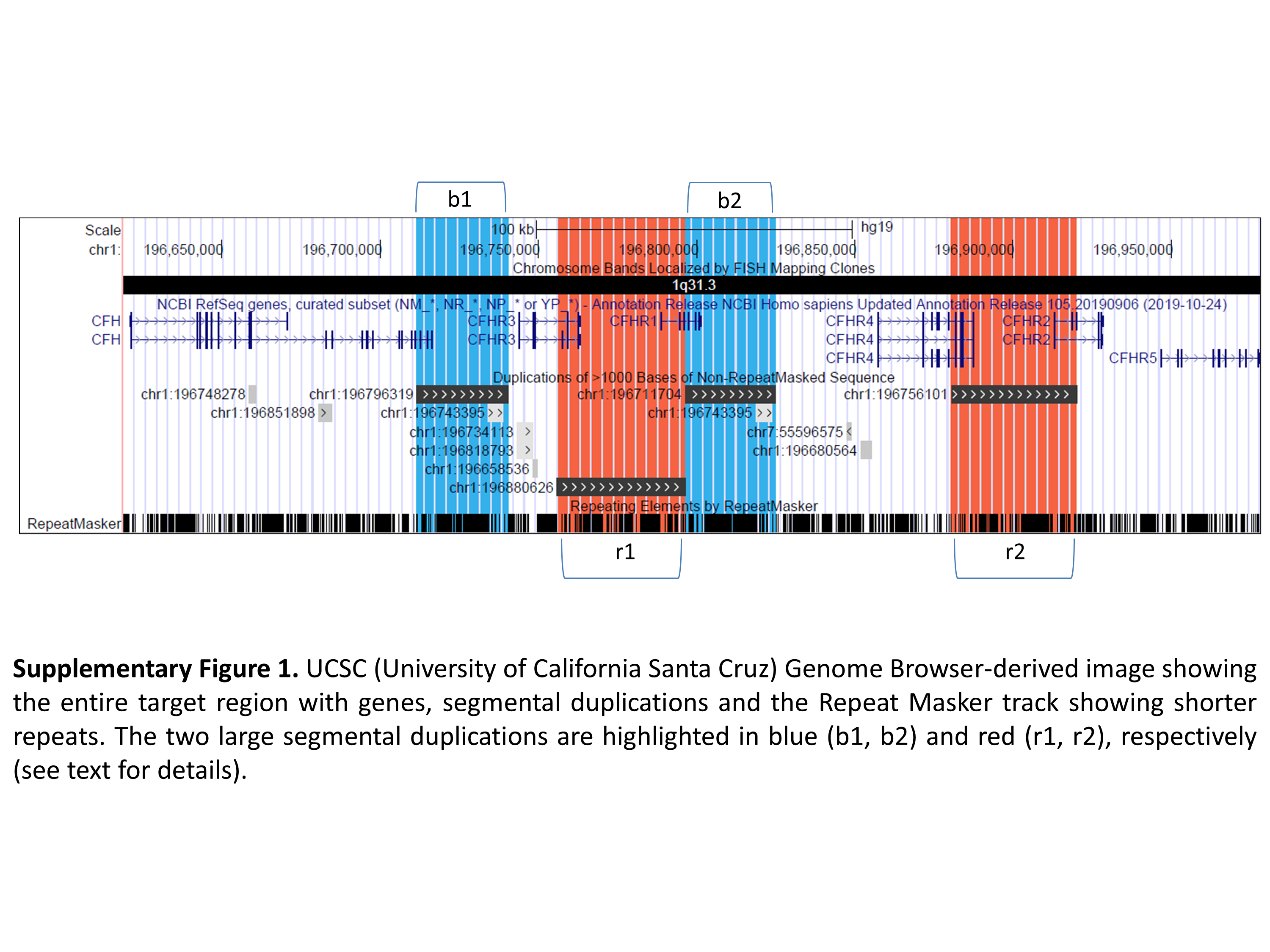

Supplement: Supplementary file 1 [file Image_1.TIF]

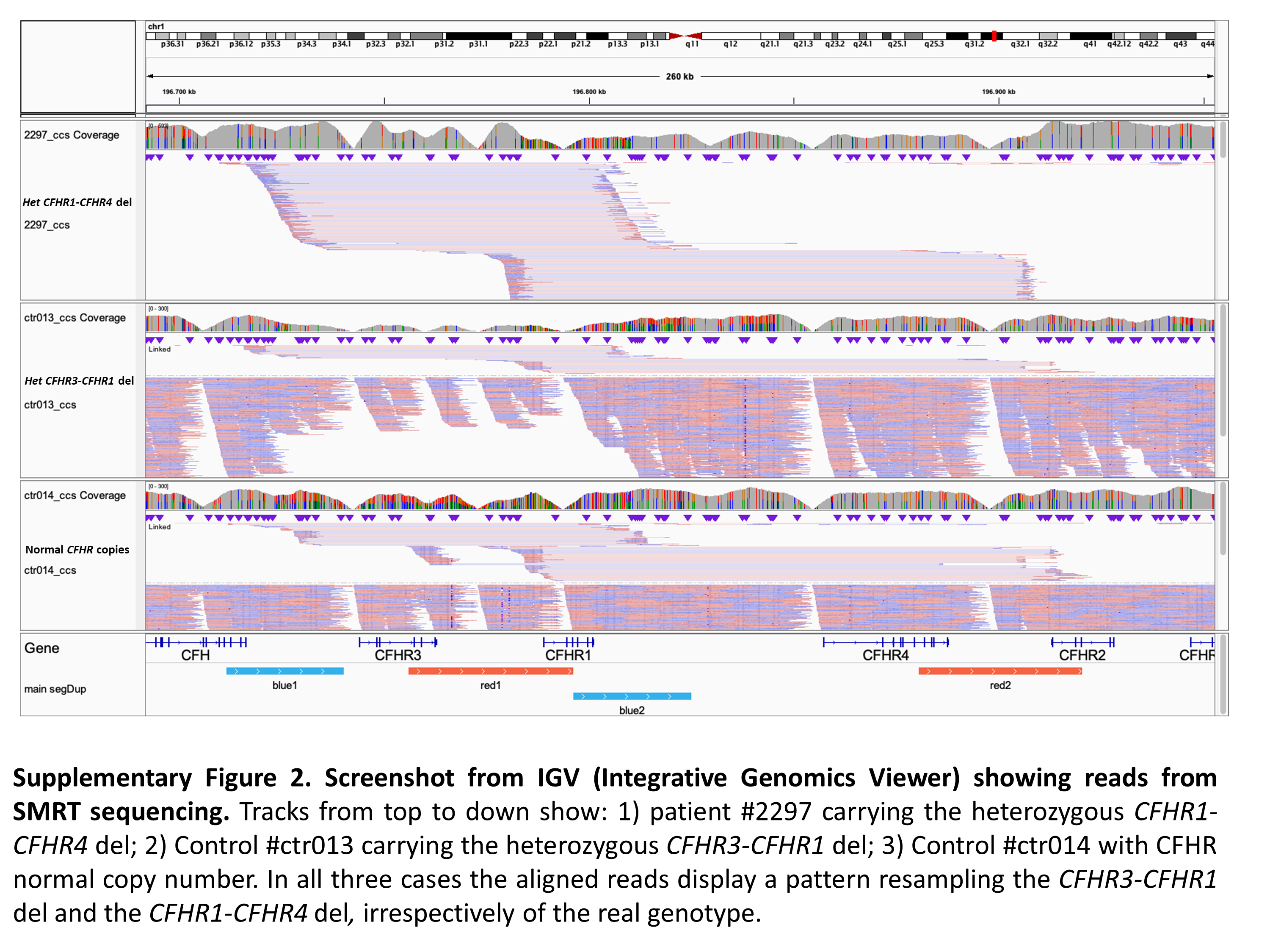

Supplement: Supplementary file 2 [file Image_2.TIF]

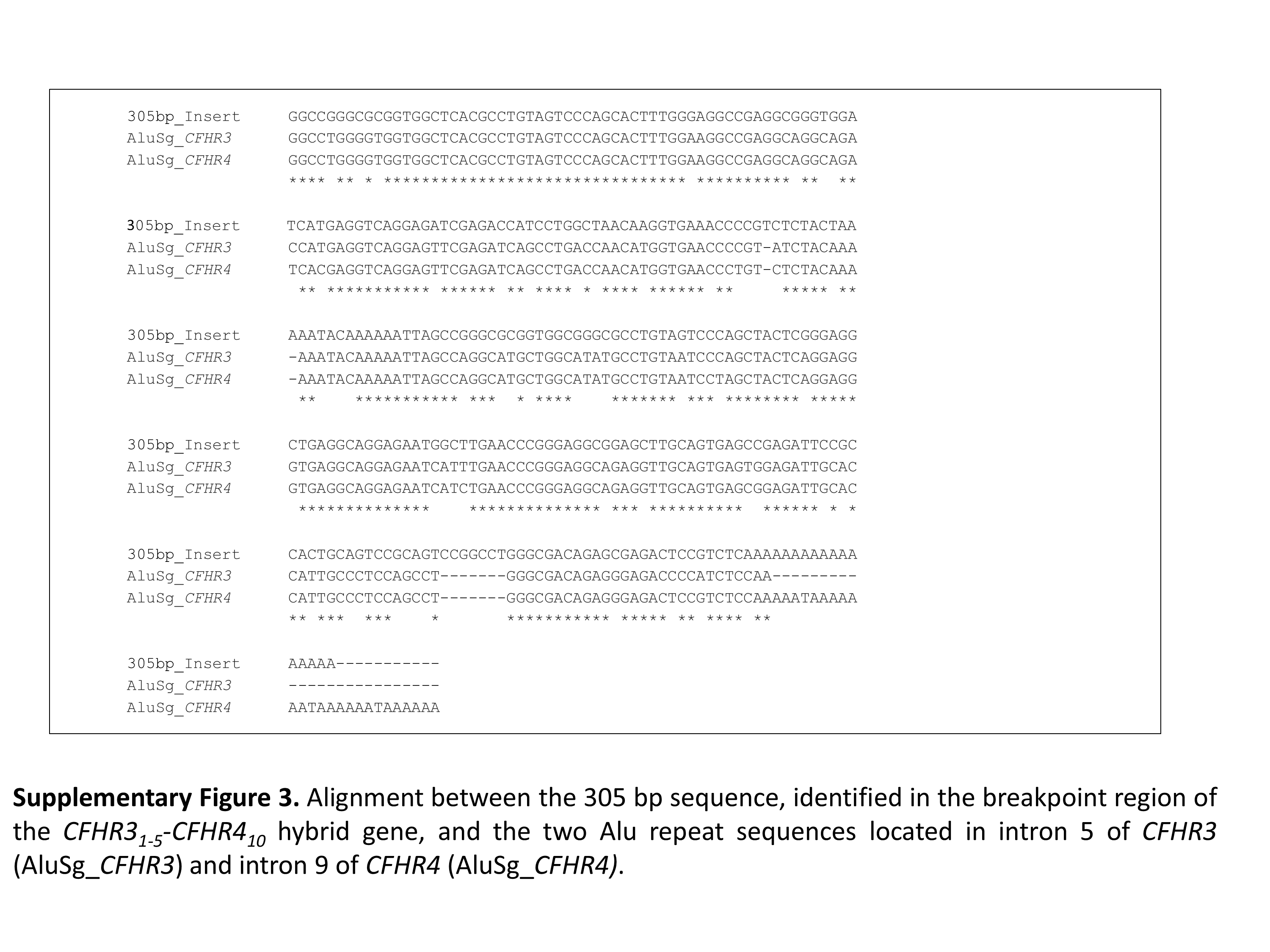

Supplement: Supplementary file 3 [file Image_3.TIF]

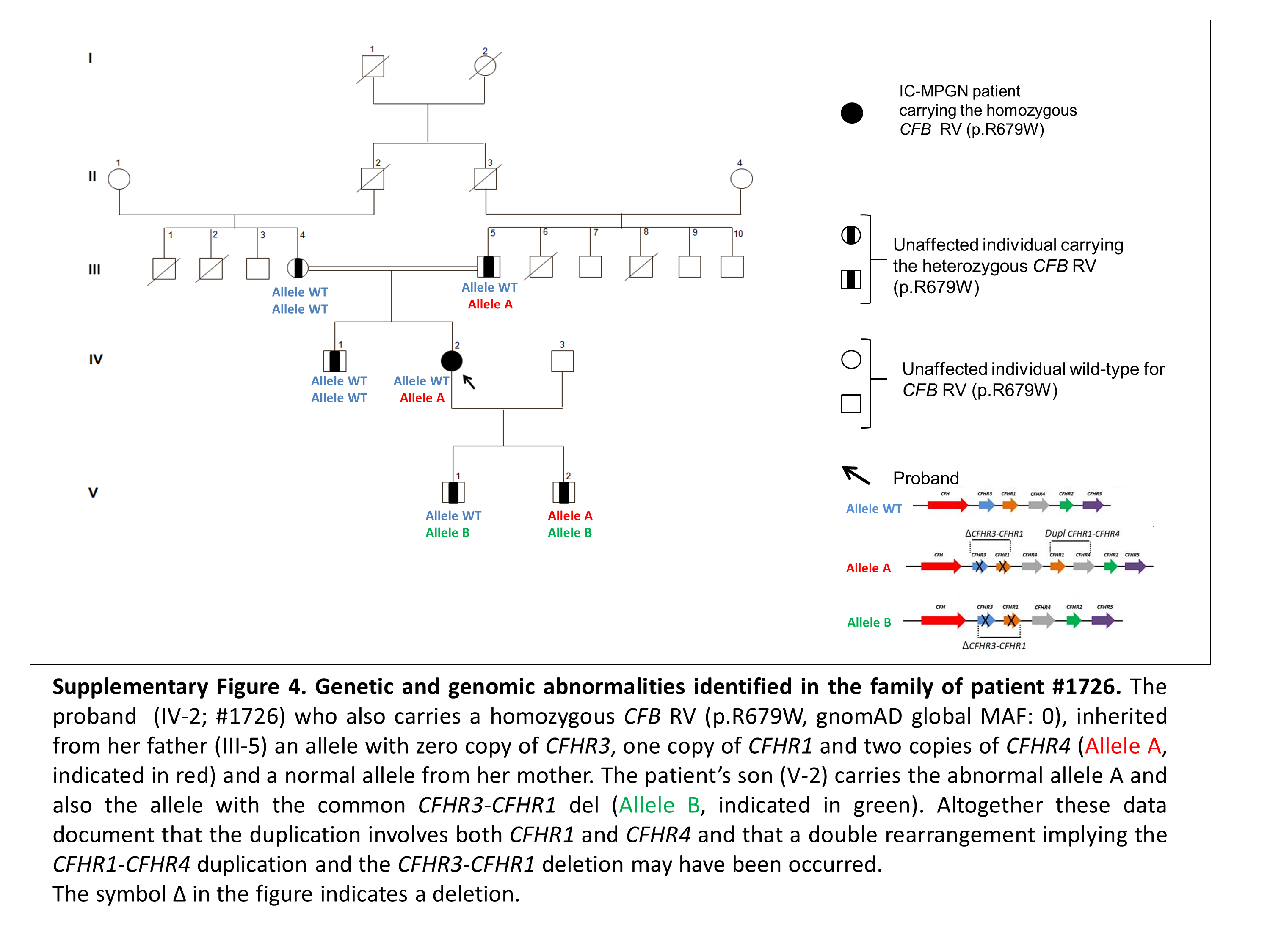

Supplement: Supplementary file 4 [file Image_4.TIF]

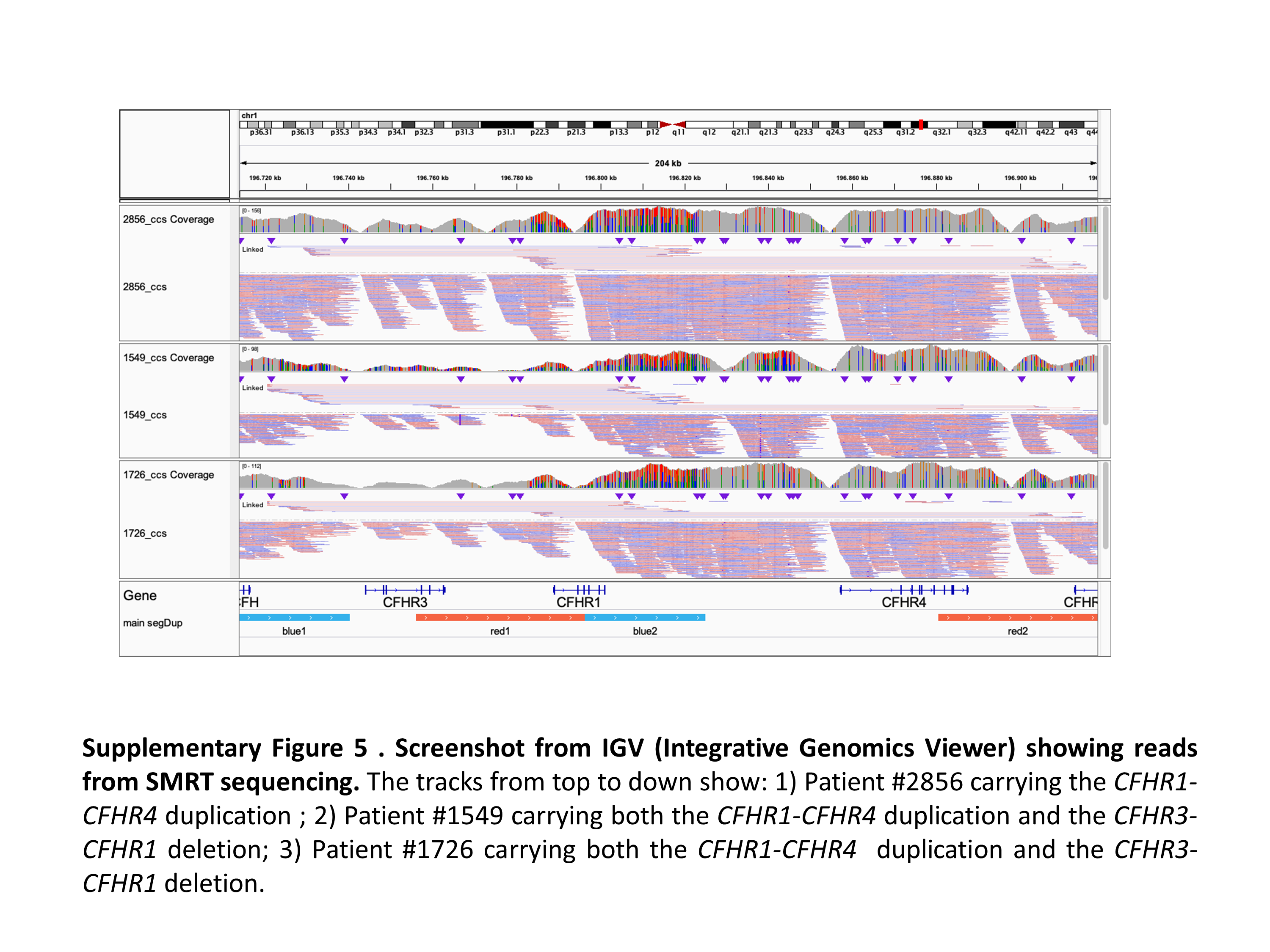

Supplement: Supplementary file 5 [file Image_5.TIF]

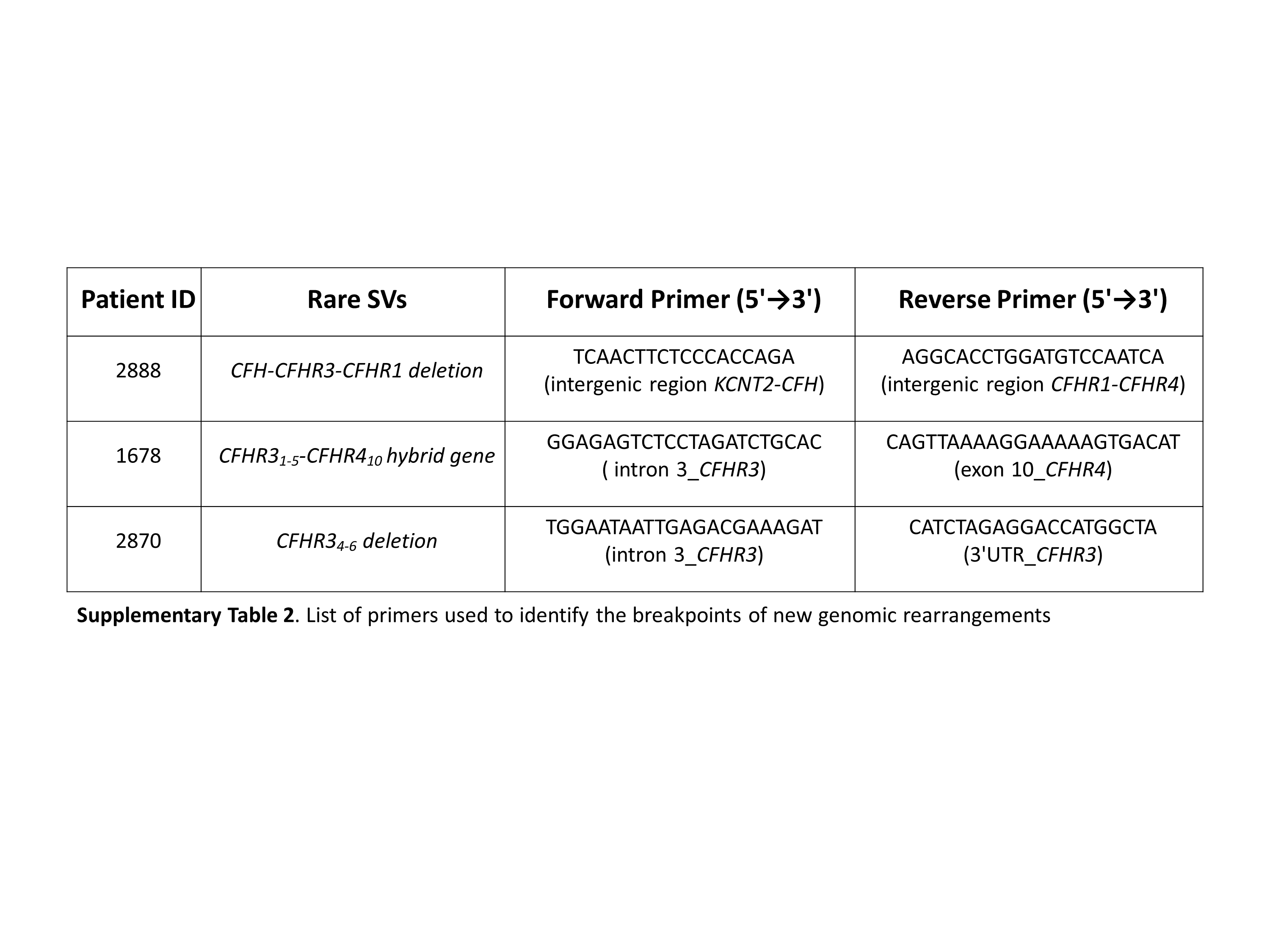

Supplement: Supplementary file 6 [file Image_6.TIF]

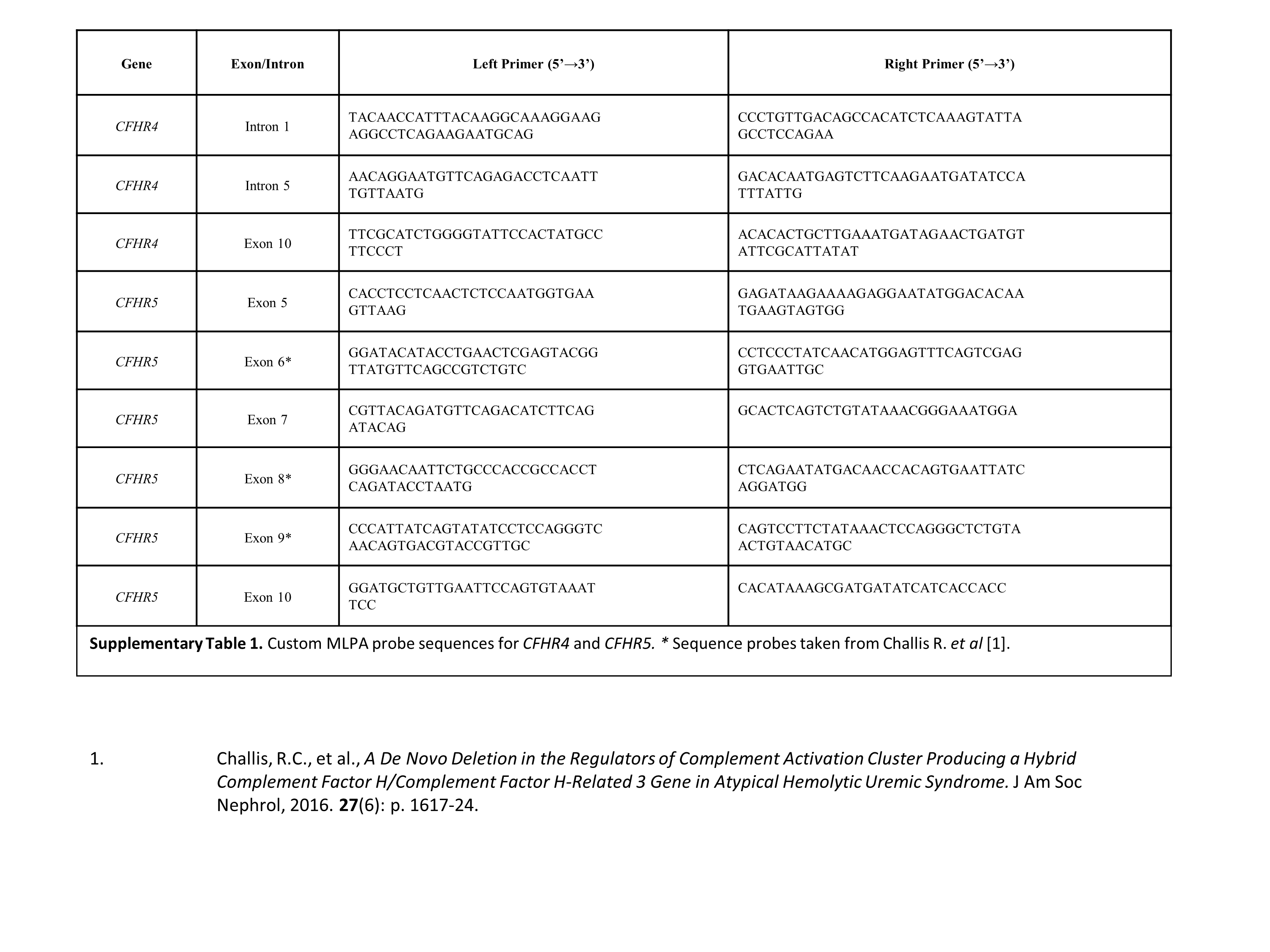

Supplement: Supplementary file 7 [file Image_7.TIF]
